# Propolis-containing formulations in caries prevention - a systematic review and meta-analysis

**DOI:** 10.1007/s00784-026-07065-6

**Published:** 2026-08-13

**Authors:** Luciana Pisenti Berto, Richard Johannes Wierichs, Letícia Oba Sakae, Samira Helena Niemeyer, Thiago Saads Carvalho

**Affiliations:** 1 Department of Restorative, Preventive and Pediatric Dentistry, School of Dental Medicine, University of Bern, Freiburgstrasse 7, Bern, 3010 Switzerland; 2 Department of Operative Dentistry and Periodontology, University of Bonn, Welschnonnenstrasse 17, 53111 Bonn, Germany

**Keywords:** Propolis, Dental caries, Meta-analysis, Systematic review, Dental plaque index

## Abstract

**Objectives:**

This systematic review and meta-analysis evaluated the clinical efficacy of propolis-containing formulations (PCFs) in preventing dental caries (PROSPERO: CRD420251246707).

**Methods:**

This study adhered to the PRISMA 2020 guidelines. Systematic database searches were conducted (December 11, 2025) without language or date restrictions. Eligible studies were randomized clinical trials (RCTs) assessing PCFs (dentifrices, mouthwashes, tablets, etc.) for caries-related outcomes compared with negative control or chlorhexidine-containing formulations (CHX). Standardized mean differences (SMD) were calculated using random-effects models. Meta-analyses were conducted for the outcomes plaque index (PI) and microbial counts in saliva or biofilm (MC). RoB 2 tool and GRADE were used as quality assessment tools.

**Results:**

Of 1,919 identified records, 45 studies were included. Direct caries-related outcomes (caries indices) were rarely assessed and unsuitable for meta-analysis. PCFs significantly reduced PI compared with negative control (SMD[95% CI]: -1.11 [-1.52, -0.70], *p* < 0.01) and showed no significant difference compared with CHX (0.46 [-0.04, 0.95], *p* = 0.07). Similar patterns were observed for MC (PFCs vs. negative control (-1.45 [-2.43, -0.48], *p* = 0.004; PFCs vs. CHX: 0.38 [-0.11, 0.87], *p* = 0.13). Certainty of evidence was very low due to high risk of bias, inconsistency and indirectness.

**Conclusion:**

Propolis-containing formulations may reduce plaque indices and oral microbial counts, suggesting a potential role in caries prevention. However, high-quality studies assessing direct caries outcomes are needed to properly address the research question.

**Clinical relevance:**

Propolis-containing formulations are widely available and well accepted by patients. Clinicians may consider them as adjuncts to oral biofilm control but the evidence directly supporting their clinical efficacy in caries prevention is still not well established.

**Supplementary Information:**

The online version contains supplementary material available at https://doi.org/10.1007/s00784-026-07065-6.

## Introduction

Effective caries prevention relies on a multi-level strategy. The daily mechanical disruption of dental biofilm through toothbrushing and flossing, along with diet modification, remain essential [1], but chemical adjuncts can enhance the preventive effect. Fluoride-based products, such as toothpastes, varnishes, and mouthwashes are the foundation of caries prevention programs [2]. Their effectiveness results from direct action on tooth mineral, preventing demineralization and promoting lesion remineralization. Dental caries, however, is a biofilm-mediated disease caused by a dysbiotic shift in the supragingival microbiota, primarily driven by frequent consumption of fermentable carbohydrates and prolonged acidic conditions [3], which favours the dominance of acidogenic and aciduric microorganisms [4]. Therefore, modern caries management aims not only at restoring tooth structure but also at rebalancing the oral microbiota [5]. In this context, chemical agents may act specifically on the biofilm, thereby promoting the stability of the microflora.

Biofilm accumulation, commonly assessed using plaque indices and microbial counts, is frequently used as an indirect indicator of caries risk, as higher plaque levels and cariogenic bacterial loads are associated with increased acid production and demineralization of the dental tissues [6–8]. Antimicrobial agents in dentifrices and mouthwashes such as chlorhexidine can reduce biofilm accumulation in high-risk individuals [9], contributing to caries reduction. However, side effects including tooth staining and taste alteration, along with challenges in patient adherence have increased interest in naturally derived alternatives [10].

Some natural agents reduce the cariogenic potential of the biofilm and favour a health-related microbial community [11]. The growing interest in such agents is reflected in both the scientific search for effective and sustainable preventive strategies as well as in the increasing consumer preference toward natural products that support green economy principles and environmentally responsible consumption [12]. Examples of these agents are polyphenols present in green tea, grape seeds, cranberries [13, 14], or propolis [11].

Propolis is a resinous material collected by bees (mainly *Apis mellifera* L.) from plant sources to protect the hive from infection and has been used for centuries in traditional medicine [15]. Its potential as a natural adjunct in oral care has been investigated in vitro [16] and in vivo [11, 17], showing promising results. Usually rich in flavonoids, phenolic acids, and other bioactive compounds, propolis has shown anti-inflammatory, antioxidant and antimicrobial properties, making it an attractive candidate for improving oral health and inhibiting the oral biofilm formation [10]. The anti-biofilm activity of propolis is largely attributed to its ability to inhibit both the expression and activity of glucosyltransferases, the key enzymes responsible for extracellular polysaccharide synthesis and biofilm assembly [11]. Apigenin and tt-farnesol are examples of propolis compounds which are effective inhibitors of these enzymes [18]. When Streptococcus species are exposed to ethanolic propolis extracts, a marked reduction in water-insoluble glucan formation is noted [19], which explains the frequently observed inhibitory effect of propolis on growth and adhesion of cariogenic microorganisms [20], indicating the potential of the substance as an adjunct in oral care products.

Due to their biological properties and natural origin, propolis-containing formulations (PCFs) are widely available [16] and generally well accepted by patients [21]. Mouthwashes, dentifrices, chewing gums and tablets containing the substance are commercialized with the prerogative of improving oral health [16] but the evidence that these formulations can actually prevent caries disease was never evaluated systematically. An evidence-based assessment of the potential role of PCFs as adjuncts in caries prevention strategies may help guide clinical decision-making, patient counselling, and future research in the field. Therefore, this systematic review and meta-analysis aimed to assess the clinical efficacy of PCFs in the prevention of dental caries.

## Materials and methods

### Protocol and registration

This review adhered to the Preferred Reporting Items for Systematic Reviews and Meta-Analyses (PRISMA) 2020 statement [22]. The protocol for the study was registered (CRD420251246707) at the International Prospective Register of Systematic Reviews (PROSPERO).

### Eligibility criteria

The research question was formulated using the PICOS framework (Population, Intervention, Comparison, Outcome, and Study design), as follows: *“Do propolis-containing formulations prevent dental caries in comparison to control?”* Studies were included if they involved human participants of any age (Population) and evaluated the use of propolis extract or propolis-containing formulations containing no other antimicrobial substance (Intervention) compared with negative control (propolis-free formulations containing no other antimicrobial substance, saline solution, distilled water, or no treatment) or chlorhexidine-containing formulations (Comparison). Outcomes directly or indirectly related to caries prevention, such as caries and plaque indexes or microbial counts in saliva or biofilm, were considered (Outcomes). Only randomized controlled trials (Study design) were included. Studies that used antimicrobial agents other than chlorhexidine as the control group were excluded.

### Search strategy

Systematic online searches were conducted in PubMed/MEDLINE, CENTRAL (Cochrane Central Register of Controlled Trials), Scopus, Embase and LILACS. The LILACS database was accessed via the Virtual Health Library (VHL) platform, which provides aggregated access to regional Latin American and Caribbean health literature. In order to further identify potential studies and grey literature, the search was also conducted in the register of clinical trials of the US National Institutes of Health (www.clinicaltrials.gov) and in Google Scholar. Searches were performed on December 11, 2025, without language or publication year restrictions. A comprehensive search strategy was developed and appropriately revised for each database aiming a broad exploration (Supplementary Material - Table S1) and combined controlled vocabulary (MeSH terms) and free-text keywords relevant to propolis and dental caries, joined by Boolean operators (“AND”, “OR”). The search strategies for PubMed/MEDLINE are shown in Table 1. Reference lists of included studies and relevant reviews were also screened to identify additional eligible studies.

**Table 1:** Search strategy used for Pubmed/MEDLINE database

Search	Query	Results
#1	propolis	5.508
#2	((((((((((dental caries) OR (decay)) OR (carious)) OR (cavit*)) OR (cariogenic)) OR (deminerali*)) OR (reminerali*)) OR (dental biofilm)) OR (dental plaque)) OR (plaque index)) OR (Streptococcus mutans)	532.949
#3	#1 AND #2	393

### Study selection

Identified records were imported into EndNote [23], and duplicates were automatedly removed. Screening was performed by two independent examiners (L.P.B. and R.J.W.), following the eligibility criteria, using the Rayyan web application [24]. Inter-examiner agreement for study selection was assessed using the Cohen’s kappa coefficient (κ). Articles were initially screened based on titles and abstracts, followed by full-text assessment for eligibility. Discrepancies were resolved through consensus-based discussion. Reviewers were not blinded to journal names, identities of the authors, institutional affiliations, or study results.

### Data extraction

Three previously calibrated reviewers (S.H.N., L.O.S. and T.S.C.) independently extracted data using a pre-defined form. During the calibration process, reviewers independently extracted data from the first five studies, and the results were compared to identify and resolve discrepancies through consensus-based discussion. Extracted information included first author, year of publication, title, study design, type and concentration of propolis, formulation type, details of the study groups, sample size, age of patients, outcomes, conclusions and data for meta-analysis (measures of central tendency and dispersion). When required, numerical results or graphical data were converted into means and standard deviations to allow the inclusion of a higher number of studies in the statistics.

### Data synthesis and statistical analysis

Meta-analyses were conducted for studies reporting similar outcomes. Experimental groups were compared with negative control or chlorhexidine groups in separate analyses. Subgroup analyses were performed by formulation type, propolis concentration and observation time in order to explore potential sources of heterogeneity. For continuous variables, the primary measures of effect between treatment and control groups were the mean differences (MD) when studies used the same outcome, or the standardized mean differences (SMD) when studies used the same construct but different scales. Using the Review Manager (RevMan) from the Cochrane Collaboration [25], a random-effects model was applied to account for anticipated heterogeneity across studies, assuming that true effect sizes vary due to differences in populations, interventions, propolis concentration, formulation type and observation time [26, 27]. Inter-study heterogeneity was evaluated by the Chi^2^ and I^2^ statistics [28]. To avoid unit-of-analysis errors, the guidelines outlined by the Cochrane collaboration (Chap. 6.2.1) were followed [29]. Therefore, when studies reported more than one follow-up period, data from a single time point were used (the longest follow-up period not exceeding 90 days). In the same way, in studies that assessed the counts of multiple bacterial species, only results for *Streptococcus mutans* (the most consistently reported microorganism among the included studies) were considered in the meta-analysis to ensure independence of observations.

### Sensitivity analyses

Sensitivity analyses excluded studies at high risk of bias and compared results from fixed- and random-effects models to assess robustness. In addition, because baseline oral health status varied across studies, a separate sensitivity analysis was performed excluding studies primarily investigating gingivitis or periodontitis to assess the influence of this clinical heterogeneity on the pooled estimates.

### Risk-of-bias and certainty of evidence

The risk of bias in the included studies was assessed independently and in duplicate by two reviewers (L.P.B. and T.S.C.) using the Cochrane RoB 2 tool [30]. Discrepancies were resolved by consulting a third reviewer (R.J.W.). Publication bias was assessed visually using funnel plots of effect size against standard error. Asymmetry in the funnel plot was considered suggestive, but not definitive, evidence of publication bias [31]. The grading of evidence for each outcome was rated following the GRADE approach [32] using the GRADEpro GDT software [33].

## Results

**Fig. 1:**
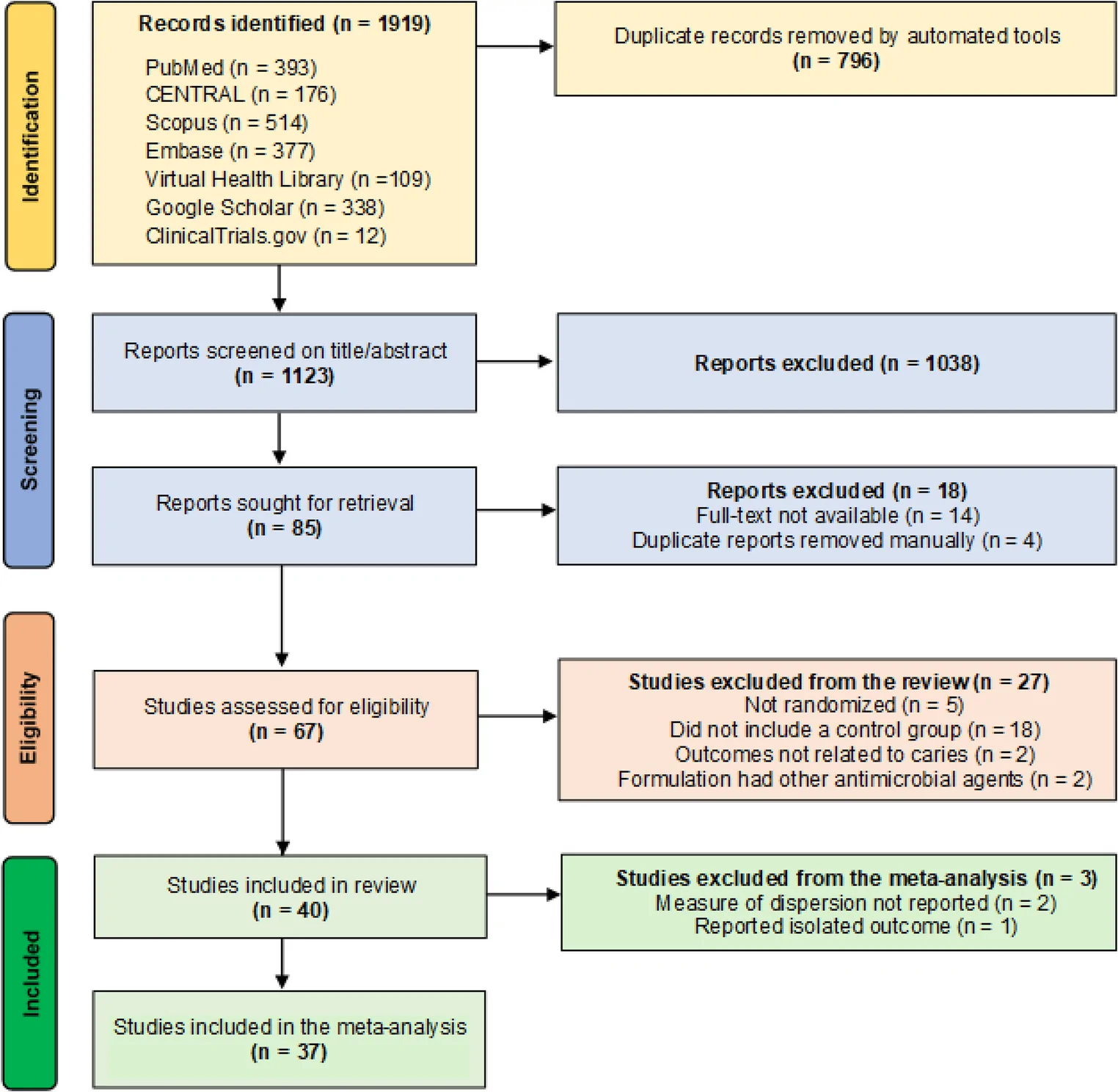
Flow chart of the study selection process (PRISMA flow diagram)

The selection process is summarized in the PRISMA flow diagram (Fig. 1). A total of 1,919 records were initially identified. After removing duplicates (*n* = 796) and after title and abstract screening, 67 studies were assessed for eligibility. After full-text screening, 27 studies were excluded either because they were not randomized (5 studies), or did not include a negative control group or chlorhexidine group (18 studies), or because they reported outcomes not related to dental caries (periodontal pocket depth, clinical attachment level − 2 studies [34, 35]), or the tested formulation had antimicrobial agents other than propolis (2 studies [36, 37]). The list of excluded studies is available in Supplementary Material - Table S2. Finally, 40 studies were included in the review and 37 in the meta-analysis. Three studies were excluded from the meta-analysis: two studies did not report measures of dispersion for the results [38, 39] and one study reported an isolated outcome, so meta-analysis was not possible [40]. Inter-reviewer agreement for the study selection was considered substantial (κ = 0.93).

Detailed characteristics of the included studies are presented in Tables 2 and 3. A total number of 1,748 patients were evaluated with ages ranging from 3 to 60 years. Among the included studies, 33 used parallel-arm design, and 7 used a crossover design.

**Table 2:** Characteristics of the included studies evaluating mouthwashes

Study (country)	Type or region of propolis	Propolis concentration	Study design	Baseline oral health information of participants	Treatment protocol	Age (years^a^)	Outcomes included in the MA	Plaque index description	Time of the evaluations	Relevant results related to propolis	NC group
Anauate -Netto et al. 2013 (Brazil) [41]	typified propolis	2%	parallel RCT	balanced number of decayed or restored teeth	1 min, 2x/day, 28 days	18–55	mutans streptococci counts	n/a	0, 7, 14, 28 and 45 days	Propolis decreased *S. mutans* but not Lactobacilli compared to NC.	placebo
Arjun et al. 2015 (India) [42]	Indian propolis	30%	parallel RCT	chronic generalized gingivitis	1 min, 2x/day, 5 days	31.2 ± 5.83 18 − 50	plaque index	Turesky-Gilmore plaque index	0 and 5 days	Propolis reduced PI compared to NC, but similar to CHX.	saline solution
Bakery et al. 2023 (Egypt) [43]	not informed	5%	parallel RCT	not informed	2x/day	12–14	plaque index and *S. mutans* counts	Silness and Löe plaque index	0, 1, 30, 60 and 90d	Propolis reduced PI compared to NC.	toothpaste without propolis
Bapat et al. 2021 (India) [44]	Indian propolis (Maharashtra)	10% (w/w, estimated)	parallel RCT	not informed	1 min, 2x/day, 3 months	18–22	plaque index and *S. mutans* counts	Silness and Löe plaque index	0, 15, 30 and 90d	Propolis reduced PI and dental caries pathogens compared to NC.	distilled water
Dehghani et al. 2019 (Iran) [45]	not informed	1%	parallel RCT	fixed orthodontics patients	2x/day, 3 weeks	19.86 ± 4.19	plaque index	Silness and Löe plaque index	0 and 22 days	Propolis reduced PI similar to CHX.	none
Dodwad and Kukreja 2011 (India) [46]	not informed	not informed	parallel RCT	chronic generalized gingivitis	1 min, 2x/day, 5 days	18–50	plaque index	Turesky-Gilmore plaque index	0 and 5 days	Propolis reduced PI compared to NC.	saline solution
Gunjal et al. 2024 (Malaysia) [47]	propolis from Malaysia (NHF)	0.2% (abstract) 5% (text)**	Latin-square crossover RCT	chronic generalized gingivitis	1 min, 2x/day, 21 days; 15 days washout	23.86	plaque index	Silness and Löe plaque index	0 and 21 days	Propolis reduced PI similar to CHX.	placebo
Guru et al. 2019 (India) [48]	commercially available propolis from India	5%	crossover RCT	DMFT < 2	single rinse, 7 days washout	8–12	S. mutans counts	n/a	before and immediately after rinsing	Propolis did not reduce *S. mutans* counts as much as CHX.	none
Koo et al. 2002 (Brazil) [18]	propolis from Southern Brazil	3%	crossover RCT	good oral health	1 min, 2x/day, 3 days; 7 days washout	26.8 ± 7.8	plaque index	Silness and Löe plaque index	3 days	Propolis reduced PI compared to NC.	placebo
Lile et al. 2025 (Romania) [49]	not informed	10%	parallel RCT	plaque index between 1.5 and 3.0	30 s, 2x/day, 4 weeks	18–50	plaque index	Silness and Löe plaque index	0 and 4 weeks	Propolis reduced PI compared to NC, but similar to CHX.	placebo
Lisbona-Gonzales et al. 2021 (Spain) [50]	Spanish propolis	2%	parallel RCT	chronic periodontitis	1 min, 3x/day, 2 days	50–60	*S. mutans* counts	n/a	2 days	Propolis reduced *S. mutans* counts compared to NC.	placebo
Murray et al. 1997 (United Kingdom) [51]	comercial product (Bee Health, UK)	10%	parallel RCT	good oral health	1 min, 2x/day, 5 days	not informed	plaque index	Silness and Löe plaque index	5 days	Propolis did not reduce PI compared to NC or CHX.	placebo
Naggar et al. 2021 (Egypt) [39]	not informed	not informed	parallel RCT	high caries risk patients (ADA caries risk assessment)	60 s, 3x/day, 7 days	17–50	study not included in the MA	n/a	7 days	Propolis reduced *S. mutans* counts compared to NC, but similar to CHX.	saline solution
Porwal et al. 2018 (India) [52]	not informed	not informed	parallel RCT	chronic generalized gingivitis	10 ml, 2x/day, 15 days	20–40	plaque index	Turesky modification of the Quigley-Hein plaque index	0, 7 and 28 days	Propolis reduced PI similar to CHX.	none
Pundir et al. 2017 (India) [53]	not informed	20%	parallel RCT	chronic periodontitis with three or more nonadjacent teeth with periodontal pockets ≥ 5 mm	1 min after SRP and 2 × 1 min after 24 h + brushing tongue + subgingival irrigation	25–55	plaque index	not informed	0, 4, 12 weeks	Propolis reduced PI compared to NC.	only SRP
Salari et al. 2023 (Iran) [54]	not informed	30	parallel RCT	chronic generalized gingivitis	10 mL, 60 s, 2x/day, 4 weeks	18–50	plaque index	O’Leary plaque index	0 and 2 weeks	Propolis did not reduce *S. mutans* counts as much as CHX.	none
Santiago et al. 2018 (Brazil) [55]	Brazilian propolis (São Paulo)	2.60%	parallel RCT	good oral health	1 min, 1x/day, 14 days	20–40	plaque index	hygiene performance index	0 and 14 days	Propolis reduced PI compared to NC, but similar to CHX.	distilled water
Seth et al. 2022 (India) [56]	not informed (commercial product)	25	parallel RCT	mild to moderate periodontitis	subgingival irrigation at baseline, 7 and 15 days	18–55	plaque index and S. mutans counts	not informed	0, 15 and 30 days	Propolis reduced PI similar to CHX.	none

^a^Data presented as range and/or mean ± standard deviation, when available

^b^Conflicting information regarding the test concentration was reported in the study

Abbreviations:* MA* meta-analysis, *RCT* randomized clinical trial, *SRP* scaling and root planning, *NC* negative control, *PI* plaque index, *CHX* chlorhexidine-containing formulations, *ADA* American Dental Association, *DMFT* Decayed, Missing, and Filled Teeth

**Table 3:** Characteristics of the included studies evaluating dentifrices and other formulations

Study (country)	Type of formulation	Type or region of propolis	Propolis concentration	Study design	Baseline oral health information of participants	Treatment protocol	Age (years^a^)	Outcomes included in the MA	Plaque index description	Observation times	Relevant results related to propolis	NC group
Abreu et al. 2000 (ref) [38]	toothpaste	red propolis	not informed	parallel RCT	not informed	10 cycles (21 days brushing + 15 days rest) during 18 months	7–10	study not included in the MA	n/a	0, 3 and 18 months	Propolis reduced caries prevalence and caries index (DMFS) compared to NC.	placebo
Aggarwal et al. 2023 (India) [57]	subgingival gel	not informed	not informed	parallel RCT	moderate to severe chronic periodontitis	topical application into the periodontal pocket after SRP	not informed	plaque index	Turesky modification of the Quigley-Hein plaque index	0, 1 and 3 months	No differences in PI between NC and propolis.	only SRP
Amano et al. 2024 (Japan) [58]	toothpaste	Brazilian green propolis	0.03%	crossover RCT	not informed	brushing 15 days; 31 days washout	18–40	plaque index	not informed	0, 1 and 2 weeks	Propolis decreased PI compared to NC.	placebo
Anani et al. 2023 (Egipt) [40]	indirect pulp capping material	propolis from Egypt	54.55% (w/w, estimated)	parallel RCT	medium to high caries risk	application after caries removal	18–50	*S. mutans* counts	n/a	0, 6 and 12 weeks	Propolis did not change bacterial counts or dentin radiopacity compared to NC.	no application after caries removal
Bhat et al. 2015 (India) [59]	toothpaste	not informed comercial dentifrice (Forever Bright^®^)	not informed	crossover RCT	not informed	1 min with NaF toothpaste + 1 min with test product; 15 days washout	19.93 ± 1.41 18 − 22	plaque index	modified gingival marginal plaque index	0 and 24 h	Propolis reduced PI compared to NC.	toothpaste without propolis (Colgate Total^®^)
Biria et al. 2019 Iran) [60]	toothpaste	not informed	not informed	parallel RCT	not informed	2x/day for 4 weeks	24–30	plaque index	modified Turesky plaque index	0 and 4 weeks	Propolis reduced PI compared to NC.	placebo
Furtado Junior et al. 2020 (Brazil) [61]	toothpaste	Brazilian red propolis (Alagoas)	1%	parallel RCT	not informed	not informed	12–16	*S. mutans* counts	n/a	0 and 28 days	Propolis decreased bacterial counts compared to NC.	commercial fluoride dentifrice
Hassan et al. 2021 (Iraq) [62]	toothpaste	not informed	not informed	crossover RCT	generalized gingivitis	2 min, 2x/day, 7 days; 7 days washout	20–35	plaque index	modified Quigley Hein plaque index	0, 24 h and 7 days	Propolis reduced PI compared to NC.	SnF_2_-containing toothpaste
Lotif et al. 2022 (Brazil) [63]	toothpaste	Brazilian red propolis	1%	parallel RCT	caries-free orthodontic patients	2 min, 3x/day, 28 days	12–18	Lactobacillus spp. counts plaque index	visible plaque index	0 and 28 days	Propolis reduced Lactobacillus spp., but not PI, compared to NC.	MFP-containing toothpaste
Machorowska-Pienidhek et al. 2013 (Poland) [64]	toothpaste	Brazilian propolis	3%	parallel RCT	uni or bilateral cleft lip and palate treated with fixed appliances	3x/day, 35 days	12.37 ± 2.28	plaque index	approximate plaque index	0 and 35 days	Propolis reduced PI compared to NC.	placebo
Machorowska-Pienidhek et al. 2016 (Poland) [65]	toothpaste	Brazilian propolis	3%	parallel RCT	oral cleft malformation treated with multibracket and removable appliances	3x/day, 35 days	9–16	plaque index	Silness and Löe plaque index	0 and 35 days	Propolis reduced PI compared to NC.	placebo
Martins et al. 2020 (Brazil) [66]	tablets	Brazilian red propolis (Rio de Janeiro)	0.80%	crossover RCT	healthy	2x/day, 7 days, 30 days washout	12.5 ± 2.93 10 − 19	Streptococcus spp. counts	n/a	7 days	Propolis did not reduce bacterial counts compared to NC.	placebo
Neto et al. 2021 (Brazil) [67]	varnish	Brazilian red propolis	2.50%	parallel RCT	caries-free, with high risk of caries (AAPD)	single application (2nd deciduous molars)	3–5.9	*S. mutans* counts	n/a	90, 180 and 360 days	Propolis did not reduce *S. mutans* counts as much as CHX.	none
Niedzielska et al. 2016 (Poland) [68]	tooth gel	Brazilian green propolis (Minas Gerais)	3%	parallel RCT	mandible stable osteosynthesis surgery, with intraoral fixation	not informed	“mature age”	plaque index	approximate plaque index according to Lange, 1986	0, 7 and 21 days	Propolis did not reduce PI compared to NC.	placebo
Prabhakar et al. 2015 (India) [69]	solution	propolis produced by honeybees (Apis mellifera)	not informed	split mouth RCT	at least three cavitated dentinal lesions in primary molars	single application, cavity desinfection for 60 s	5–12	total microbial counts	n/a	before and after caries removal and post cavity disinfection	Propolis reduced bacterial counts compared to NC.	distilled water
Raman et Jain 2023 (India) [70]	toothpaste	not informed	not informed	parallel RCT	health	1 min with NaF toothpaste + 1 min with test product	22.76 ± 1.44 24 − 30	plaque index	modified gingival marginal plaque index	0 and 24 h	Propolis reduced PI compared to NC.	toothpaste without propolis (Pepsodent^®^)
Ranjan et Anshuman 2023 (India) [71]	toothpaste	not informed	not informed	parallel RCT	health	1 min with NaF toothpaste + 1 min with test product	20.82 ± 1.48 24 − 30	plaque index	modified gingival marginal plaque index	0 and 24 h	Propolis reduced PI compared to NC.	toothpaste without propolis (Pepsodent^®^)
Sahu et al. 2023 (India) [72]	subgingival nanoparticle solution	Indian propolis (Odisha)	equivalent to 1.08%	parallel RCT	generalized periodontitis	subgingival application + sealing the pockets with cyanoacrylate	Propolis: 49.90 ± 11.63 NC: 50.10 ± 12.21	plaque index	Silness and Löe plaque index	0, 1, 3 months	Propolis reduced PI compared to NC.	saline solution
Silva et al. 2024 (Brazil) [73]	toothpaste	Brazilian red propolis (Alagoas)	1%	parallel RCT	caries-free, using fixed orthodontic appliances	2 min, 3x/day, 28 days	12–18	plaque index	not informed	0 and 28 days	Propolis reduced PI compared to NC.	MFP-containing toothpaste
Soekanto et al. 2018 (Indonesia) [74]	candy	not informed	3.87% (w/w, estimated)	parallel RCT	caries and caries-free groups	2x/day, 7 days	17–23	*S. mutans* counts of caries-free group	n/a	0 and 7 days	Propolis reduced *S. mutans* counts compared to NC.	placebo
Tanasiewicz et al. 2012 (Poland) [75]	toothpaste	Brazilian propolis	3%	parallel RCT	Healthy group and group with risk for periodontitis	not informed	not informed	plaque index of the healthy group	approximate plaque index according to Lange, 1986	0, 1 and 8 weeks	Propolis reduced PI compared to NC.	placebo
Uday Mohan et al. 2016 (India) [76]	gel	Brazilian green propolis (Uniflora^®^)	not informed	parallel RCT	primary molars with cavitated occlusal dentinal caries	application after caries removal	not informed	total microbial counts	n/a	after caries removal and after cavity disinfection	Propolis reduced microbial counts similar to CHX.	none

^a^Data is presented as range and/or mean ± standard deviation, when available

Abbreviations: *MA* meta-analysis, *RCT* randomized clinical trial, *NC* negative control, *PI* plaque index, *CHX* chlorhexidine-containing formulations, *MFP* monofluorophosphate, *AAPD* American Academy of Pediatric Dentistry

Regarding formulation type (Tables 2 and 3), propolis was most frequently tested in mouthwashes (18 studies), followed by dentifrices (14 studies). Three studies formulated propolis as a solution or paste applied to the dentin after cavity preparation [40, 69, 76], two incorporated propolis into solid preparations such as candies or tablets [66, 74], and two studies evaluated a gel or solution for subgingival application [57, 72]. Only one study incorporated propolis in a varnish for application on dental enamel [67].

With respect to the propolis origin, 13 studies evaluated products from Brazil, four from India, and one study each from Malaysia, Egypt, and Spain; however, 20 studies did not report the origin of the tested product. The type of propolis was rarely specified: only six studies reported using Brazilian red propolis, while three others used Brazilian green propolis. Propolis concentrations in the tested formulations ranged from 0.03% to 54.55%, although 12 studies did not report the concentration used (Tables 2 and 3).

Regarding the observation times, three studies assessed outcomes immediately after product application [69, 76, 77]. Among studies that evaluated outcomes following the daily use of the formulation, the mean longest follow-up period was 24.55 ± 22.53 days. In contrast, nine studies applied the product in a single or intermittent manner [40, 53, 56, 57, 59, 67, 70–72] with the longest follow-up ranging from 1 to 90 days.

Studies that evaluated plaque index described different methods for the assessment. Specifically, eight studies used the Silness and Löe plaque index, six used the Turesky-Gilmore modification, and two used the Lange approximal plaque index. In addition, one study each used the O’Leary plaque index, the hygiene performance index, the visible plaque index, and the gingival margin plaque index. However, no methodological details were provided for the latter three indices (Tables 2 and 3).

**Fig. 2:**
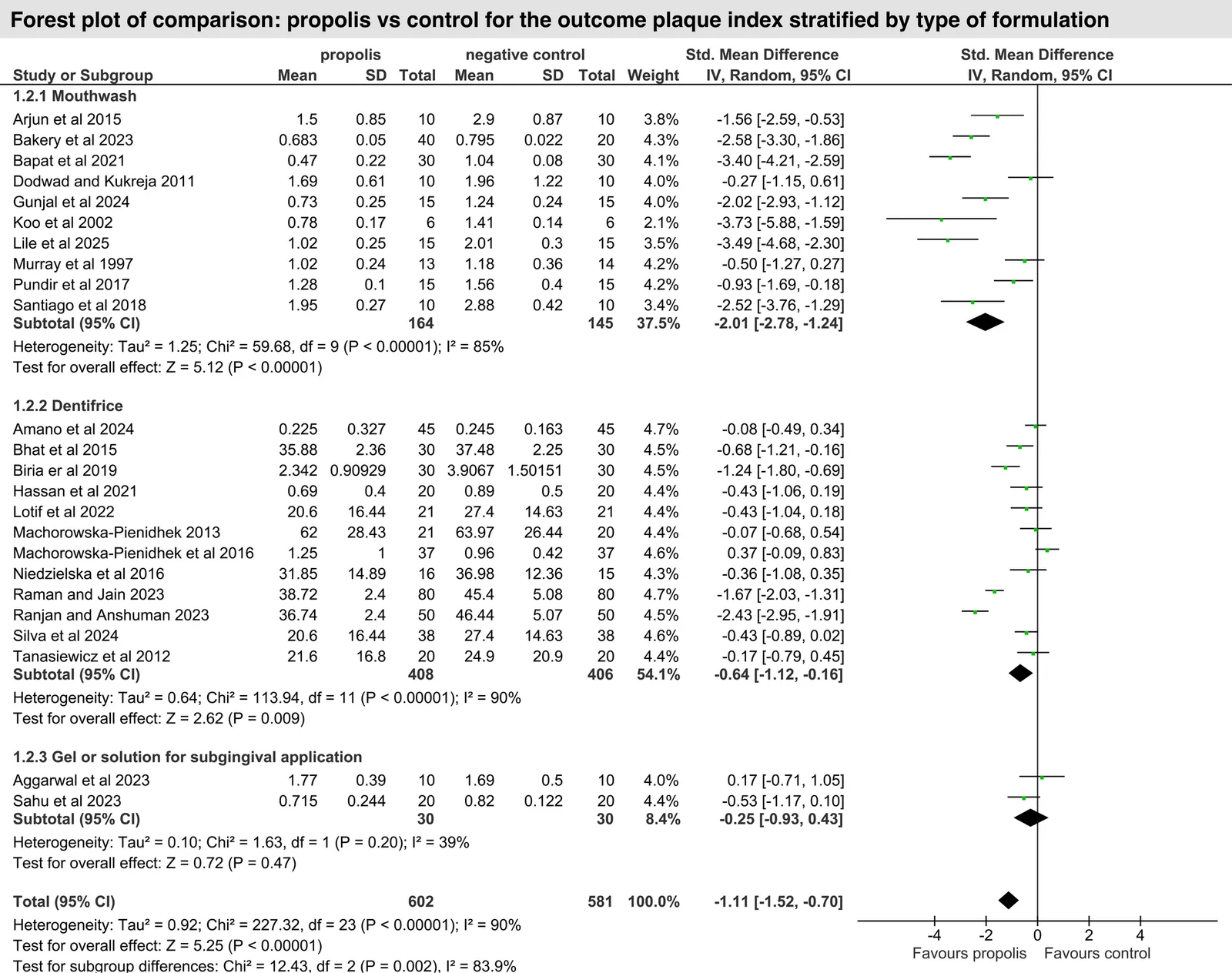
Meta-analysis for the outcome plaque index comparing propolis-containing formulations with a negative control. Studies were stratified into subgroups according to the formulation type

**Fig. 3:**
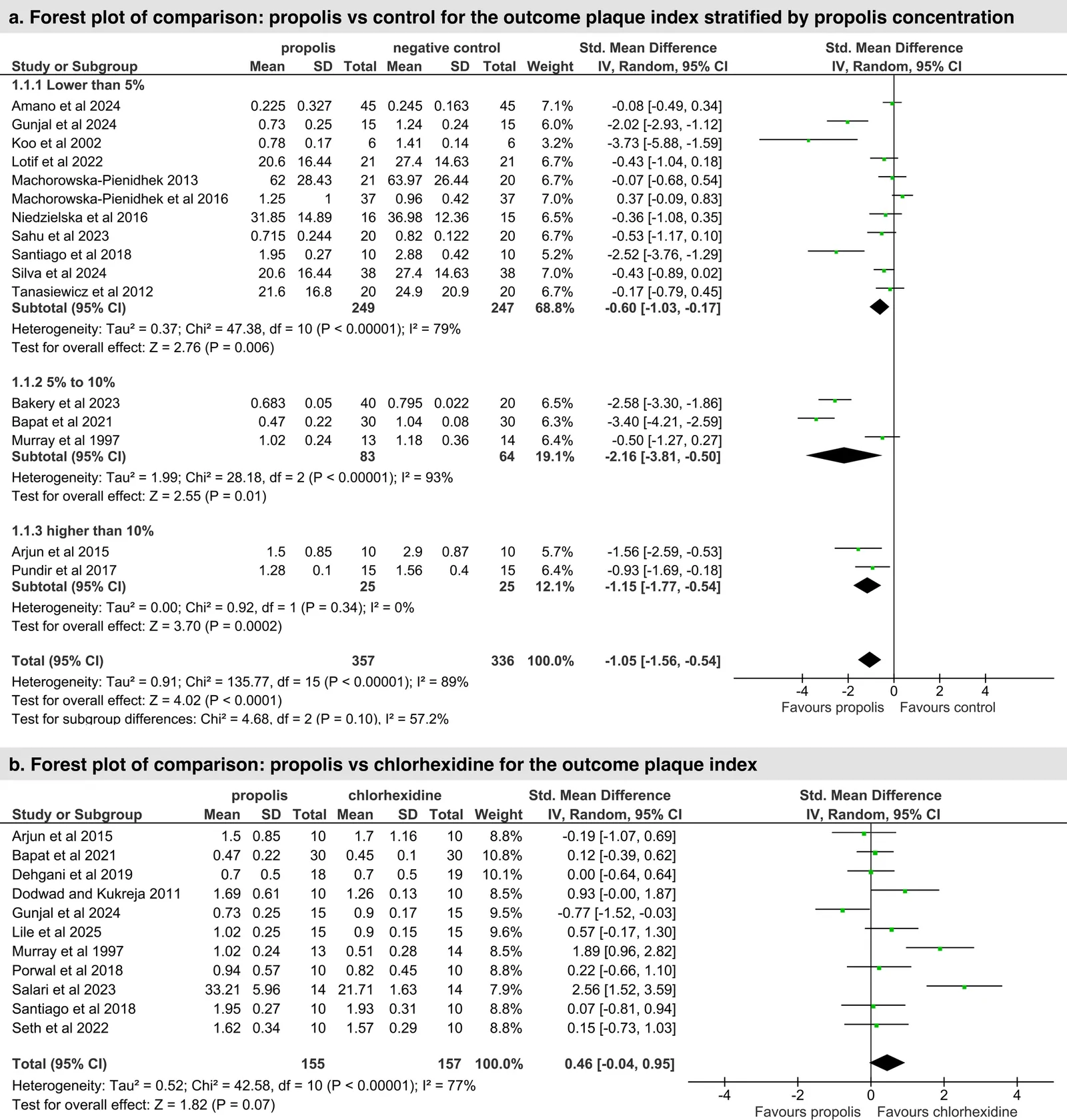
Meta-analysis for the outcome plaque index comparing propolis-containing formulations with a negative control (**a**) and with chlorhexidine (**b**). Studies were stratified into subgroups according to propolis concentration

**Fig. 4:**
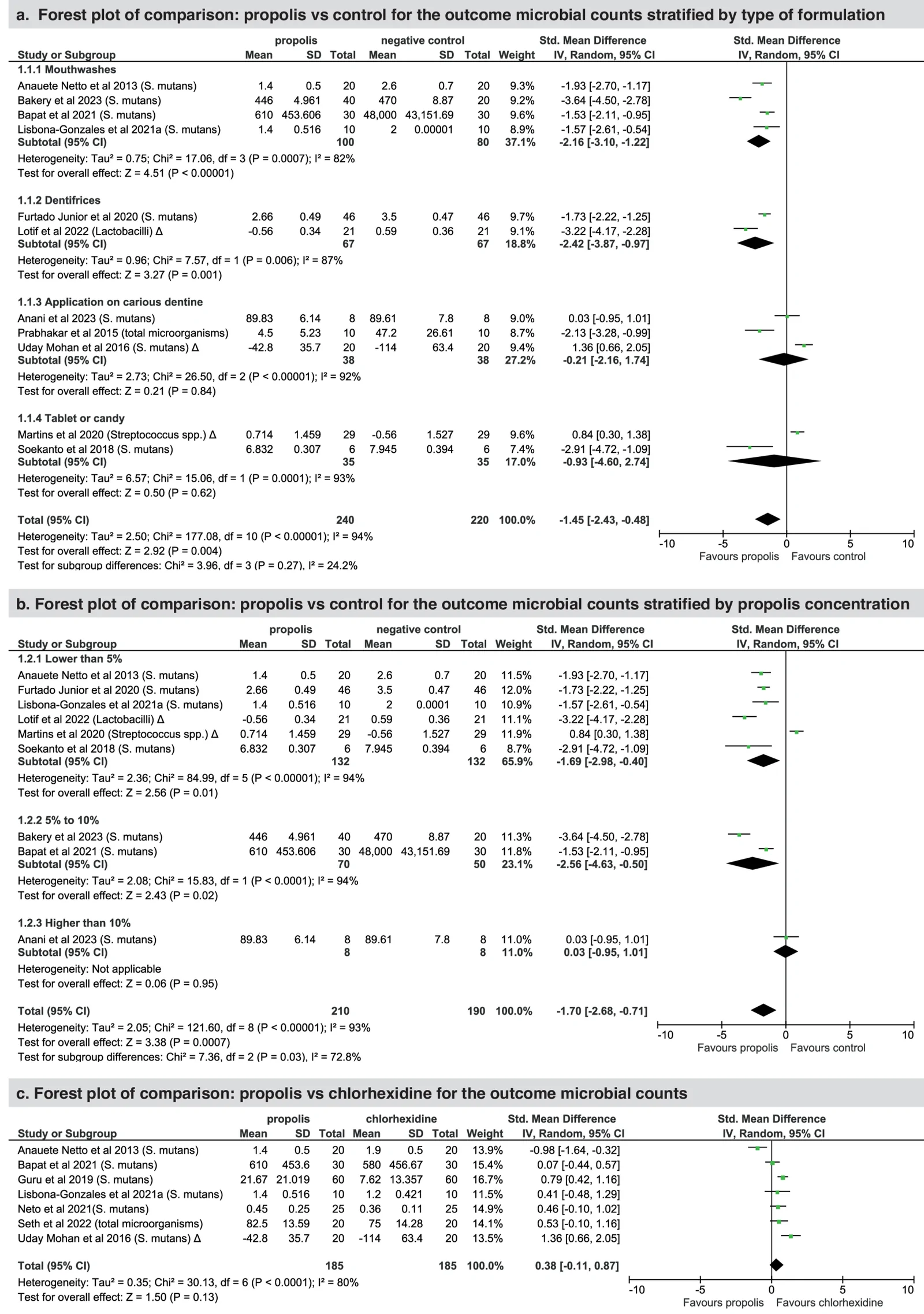
Meta-analysis for the outcome microbial counts (results in colony forming units/ml), comparing propolis-containing formulations with a negative control (**a **and **b**) and with chlorhexidine (**c**). Studies were stratified into subgroups according to the formulation type (**a**) and propolis concentration (**b**). The evaluated microorganism is shown in parentheses. “Δ” indicates that the study reported results as the difference between baseline and post-treatment; these values were multiplied by − 1 to align the direction of effect with the other studies

Meta-analyses were performed for the most frequently reported indirect outcomes related to caries prevention: plaque index (28 studies - Figs. 2 and 3) and oral microbial counts (14 studies - Fig. 4). Four included studies focused on periodontal research [53, 56, 57, 72] and were analyzed since they reported plaque index results. The following outcomes directly related to caries were also reported: DMFS (Decayed, Missing, and Filled Surfaces) and caries prevalence [38], and variation in dentin radiodensity after topical application of propolis on deep dentine [40]. However, since no other study reported these outcomes, the meta-analysis was not possible. An additional study evaluated the counts of *S. mutans* after the use of mouthrinse containing propolis, but it was excluded from the meta-analysis, since no standard deviations of the means were reported [39]. Detailed information about these studies, including their main conclusions, are shown in Tables 2 and 3.

PCFs significantly reduced plaque index compared with negative control (SMD [95% CI]= −1.11 [−1.52, −0.70], *p* < 0.01) and showed no significant difference compared with chlorhexidine-containing formulations (SMD [95% CI] = 0.46 [−0.04, 0.95], *p* = 0.07). Similar patterns were observed for microbial counts for both comparisons (PFCs vs. negative control: SMD [95% CI]= −1.45 [−2.43, −0.48], *p* = 0.004; PFCs vs. chlorhexidine: SMD [95% CI] = 0.38 [−0.11, 0.87], *p* = 0.13).

The effect of mouthwash formulations on the plaque index (SMD [95% CI]= −2.01 [−2.78, −1.24]) appeared larger than that of dentifrices (SMD [95% CI]= −0.64 [−1.12, −0.16]). The subgroup analysis by formulation type revealed that this difference was significant (Chi^2^=12.43, *p* = 0.002). The differences between these two formulations were more subtle for the outcome microbial counts (SMD_mouthwashes_ [95% CI]= −2.16 [−3.10, −1.22] and SMD_dentifrices_ [95% CI]: −2.42 [−3.87, −0.97], with no difference in the subgroup analysis (*p* = 0.27). The application of propolis on carious dentin or the use of propolis in candys or tablets did not reduce the microbial counts (*p* = 0.84 and 0.62, respectively) and the subgingival application of a PCF did not affect the plaque index (*p* = 0.47).

Subgroup analysis according to concentration range suggested a concentration-dependent effect for both outcomes, with greater effects observed at higher concentrations. However, statistically significant differences between subgroups were identified only for microbial counts (*p* = 0.03; Fig. 4b). In contrast, subgroup analyses based on follow-up period did not reveal significant subgroup differences (*p* > 0.05), indicating that observation time did not substantially influence the pooled effect estimates for either plaque index or microbial counts when compared with negative control or chlorhexidine (Supplementary Material S1-S4).

After excluding studies with high risk of bias, the sensitivity analysis generally confirmed the effect estimates and robustness of the main results (Supplementary Material - Fig. S5, S6 and S8). For microbial counts, however, the p-value for the overall effect increased (from 0.004 to 0.15) resulting in a loss of statistical significance for the comparison of propolis versus negative control (Supplementary Material - Fig. S7). After excluding studies primarily investigating gingivitis or periodontitis from the meta-analyses, the pooled effect estimates, p values for the overall effects and heterogeneity remained largely unchanged across all comparisons (Supplementary Material - Table S3).

**Fig. 5:**
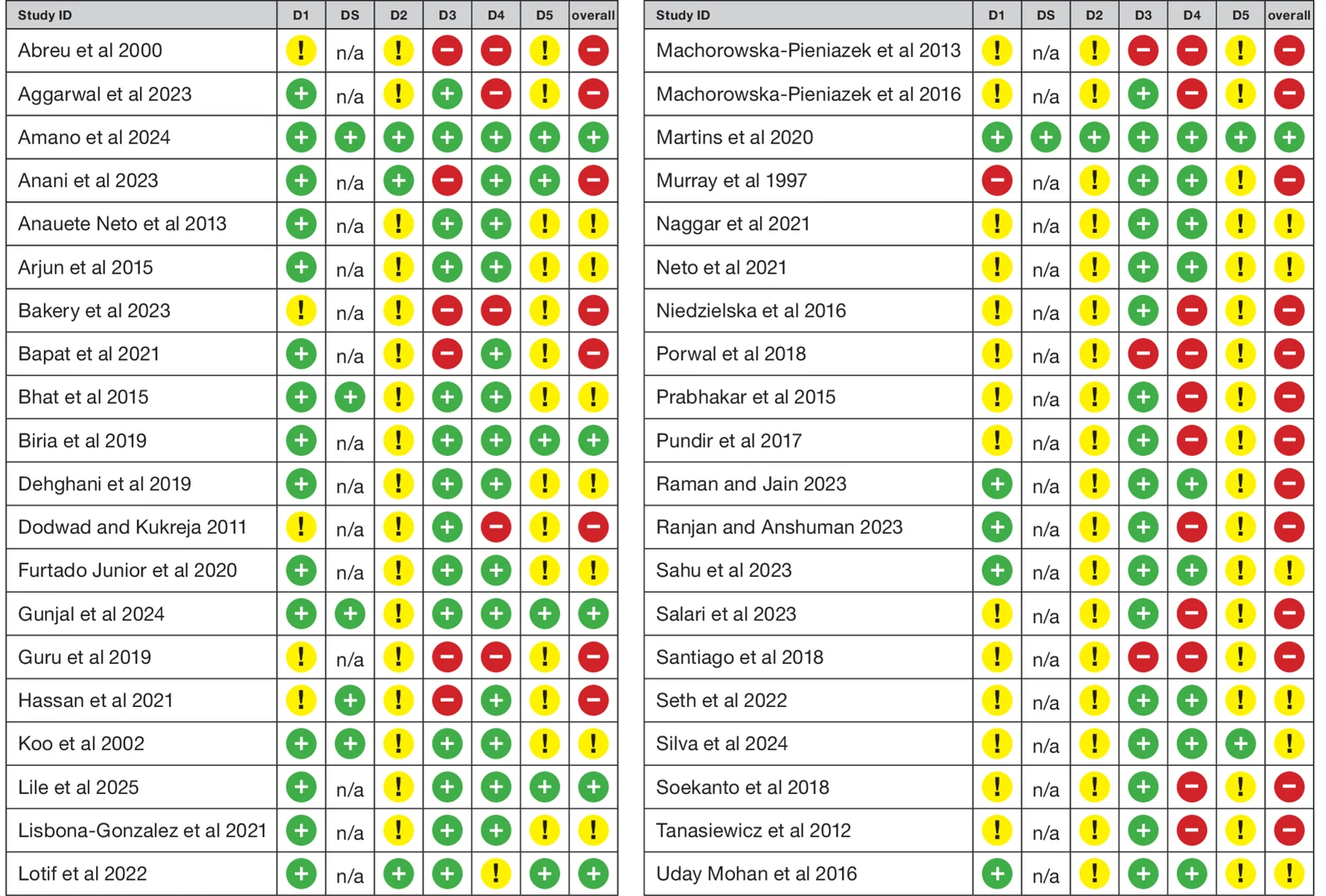
Risk of bias assessment of the studies included in the review. Risk levels are indicated by colour coding: low risk (green), some concerns (yellow), and high risk (red). Columns represent the assessed risk-of-bias domains: randomisation process (D1); bias arising from period and carryover effects (DS, applicable to crossover studies); deviations from intended interventions (D2); missing outcome data (D3); measurement of the outcome (D4); selection of the reported result (D5); and the overall risk of bias for each study. N/A indicates domains not applicable to the study design

Risk of bias assessment indicated that approximately 53% of the studies included in the meta-analysis had high risk, 32% had moderate risk, and 15% had low risk of bias (Fig. 5). The most frequent issues identified were insufficient information on randomization, blinding, and the risk of outcome selection, as most of the included studies had not previously registered a trial protocol in a public database. Certainty of evidence analyzed using GRADE was rated as very low due to high risk of bias, inconsistency and indirectness of the available outcomes in answering the research question (more information is available in Supplementary Material - Table S4). Substantial heterogeneity was observed across all comparisons, with I² values ranging from 77% to 94%. Visual analysis of the funnel plots from each comparison did not show relevant asymmetry, suggesting low risk of publication bias (data available in Supplementary Material – Fig. S9).

## Discussion

This systematic review suggests that PCFs may be effective in reducing plaque index and cariogenic microbial counts in saliva and oral biofilm. Furthermore, our findings indicate that the efficacy of PCFs is not significantly different compared with the efficacy of chlorhexidine. Thus, the results support the potential use of propolis as an additive to oral care products aiming at caries prevention, specifically at the reduction of the oral biofilm, although these results should be interpreted with caution due to the substantial heterogeneity across studies and the very low certainty of the evidence. Additionally, the included studies rarely assessed direct information on the reduction of caries lesions. The only study evaluating these outcomes observed reductions in both caries prevalence and caries index (DMFS) in children after 3 and 18 months of use of a propolis-containing dentifrice [38]. Moreover, the effects of propolis on enamel demineralization in vitro [78] and caries reduction in animal models [11, 17] have already been observed, and combined to our findings, suggest that propolis has a potential role in the prevention of caries disease.

The anti-biofilm activity observed in the PCFs can be explained by the additive effect of the compounds found in the composition of propolis. The chemical profile of propolis varies depending on the location and season of collection [79, 80], but it is generally rich in bioactive phytochemicals, particularly flavonoids and other phenolic compounds that are associated with antimicrobial properties [78], such as (3 S)-neovestitol and (3 S)-vestitol (both isoflavonoids), isoliquiritigenin (a chalcone-type flavonoid), apigenin (flavonoid), and cinnamic acid (phenolic compound). These compounds have shown antimicrobial activity against cariogenic microorganisms [11, 17, 78, 79, 81] and oral multispecies biofilms in vitro [10]. Similarly, our review indicates that these effects can also lead to biofilm reduction in patients. However, the kind of phenolic compounds, their concentrations, and ultimately the anti-biofilm effect of the PCF will depend on the kind of propolis used.

The studies showed considerable variability in terms of tested formulations, patient populations, propolis type and concentration, follow-up duration, and outcome assessment methods. These differences likely contributed to the high heterogeneity observed across all meta-analyses and may limit the interpretability and generalizability of the pooled results. Subgroup and sensitivity analyses were performed to explore this issue; however, heterogeneity remained high in most comparisons, suggesting that it is likely multifactorial and not attributable to a single variable. Similar challenges related to substantial heterogeneity have also been reported in previous meta-analyses across different dental research fields [26, 82, 83], indicating that variability across study designs and methodologies represents a broader limitation of the available evidence rather than a finding unique to the present review.

Variations in propolis composition are also an important limitation when interpreting the present findings. Propolis cannot be considered a standardized product, as its chemical composition and, consequently, its biological activity vary according to geographical origin, botanical sources, harvesting period, and extraction methods. These factors influence the concentration and profile of bioactive compounds, such as flavonoids and phenolic acids, which are responsible for its antimicrobial and anti-biofilm effects [79, 80]. Since information on propolis’ origin, extraction methods, and particularly on its chemical profile were frequently missing in the papers, their impact on the effects of PCFs could not be assessed. Therefore, the variability in propolis composition must be considered when interpreting the results and may have contributed to the observed heterogeneity.

Although differences in propolis composition limit direct comparisons, our review suggests that the different types of formulations may have different impact on the evaluated outcomes. When used as a mouthwash, propolis appear to show the best results for plaque index reduction, compared to a negative control. When added to dentifrices, however, the effect on plaque index seems lower than with mouthwashes. The difference can be explained by the effect of the mechanical biofilm removal of the toothbrush overpowering the effect of the dentifrice composition on the biofilm itself [84]. This highlights the importance of effective brushing instructions to the patient aiming at oral biofilm control. Also, mouthwashes are usually applied after the patients have brushed their teeth, and most frequently they are not washed out with a final water rinsing. As a result, active compounds in the mouthwashes are available in the oral cavity for a longer period producing a higher effect than when the dentifrice is applied during the brushing procedure, which frequently is followed by a final rinsing with water. Additionally, propolis-containing mouthwashes and dentifrices might have an additive effect in biofilm control when both products are used together, although this association was not investigated by any of the studies.

The included studies evaluated different propolis concentrations and reported distinct effect sizes, suggesting that concentration may influence the clinical efficacy of propolis-containing formulations. Since higher concentrations are expected to contain greater amounts of bioactive compounds, particularly flavonoids and phenolic substances associated with antimicrobial activity, a stronger anti-biofilm effect would be expected. In our meta-analysis, studies testing concentrations from 5 to 10% appear to show greater biofilm reduction compared with those using lower concentrations (Figs. 3a and 4b). These findings suggest a possible dose–response effect, which would be reasonable if the propolis used across studies had comparable compositions. However, because the chemical profiles were not reported and substantial inter-study heterogeneity was present, this interpretation should be approached with caution. Only one study evaluated a propolis concentration above 10% (estimated at 54.55 wt%) for microbial reduction with no difference compared with the negative control, which does not support a possible dose-response trend [40]. However, this study quantified microbial levels in deep dentine after caries removal, whereas all other studies measured bacteria in saliva or dental biofilm, which may explain the different results. In addition, propolis concentrations higher than 10% seemed to reduce less the plaque indices when compared to 5–10% concentrations. Nevertheless, the few studies that tested the highest concentrations [42, 53] evaluated patients with chronic generalized gingivitis or periodontitis. In these disease-states, the oral microbiota in dysbiosis may have higher resistance and lower response to a chemical agent [85], which explains the relatively smaller effect observed.

Studies involving patients with generalized gingivitis or periodontitis were included in this review because our eligibility criteria considered outcomes directly or indirectly related to caries prevention, with plaque index and microbial counts considered relevant indirect outcomes. Although the initial oral condition of the patients may influence study results [85], the sensitivity analysis excluding these studies did not substantially alter the pooled effect estimates, the statistical significance or the heterogeneity of the overall comparisons. These findings suggest that the inclusion of periodontal populations did not drive the main results. However, differences in baseline oral conditions remain a potential source of clinical variability. Notably, some studies did not report the baseline oral health status of the participants, which may have contributed to the observed heterogeneity.

Differences in follow-up periods among the included studies is another factor that may have contributed to the heterogeneity observed in the meta-analyses. Although subgroup analyses according to observation time suggested that follow-up duration did not substantially influence the statistical significance of the findings, the studies varied considerably regarding treatment duration and assessment periods. Moreover, a trend toward a reduction in the effect of propolis on plaque index over time was observed.

Another source of heterogeneity raised from the differences in the treatment protocols. For instance, treatment frequency ranged from a single application to three times a day, while some studies did not report the treatment frequency at all [61, 68, 75]. Moreover, the total duration of the treatment and, consequently, the period between the baseline and the final assessment varied markedly, from immediately after treatment up to several weeks. These discrepancies in exposure likely influenced the antimicrobial efficacy of propolis, contributing to different effects. Indeed, in vitro evidence indicates that the antimicrobial activity of propolis depends not only on concentration but also on treatment time [15].

Regarding the assessment of plaque accumulation, different indices were used across studies, including Silness and Löe (1964) [86], O’Leary (1972) [87], Lange’s approximal plaque index [87] and Turesky–Gilmore modification of the Quigley–Hein index [88]. Variation in plaque indices parameters likely contributed to differences in study results since each index quantify plaque in a distinct manner and with different sensitivity [89]. For instance, while Silness & Löe and Turesky–Quigley–Hein quantify plaque thickness or surface coverage respectively, the O’Leary index records only its presence or absence. As a result, the first indices detect subtle changes more effectively than the later, leading to potential discrepancies in the measured outcomes even when similar interventions are applied. Similarly, microbial counts were measured either in saliva or directly in dental biofilm samples. Since bacterial density and composition can differ substantially between these sample types [90], the choice of sampling method could influence the magnitude of observed antimicrobial effects. Such methodological differences make direct comparison between studies more challenging.

Interestingly, our meta-analysis shows that propolis has no significant difference in performance compared to chlorhexidine-containing formulations in both plaque index and oral microbial count reduction, appearing to be a reasonable alternative for biofilm control in patients with high risk of caries. Medium- and long-term use of chlorhexidine has been frequently associated with tooth and tongue staining, mucosal discoloration, alterations in taste perception, a burning sensation and genotoxic effects on buccal epithelial cells, which often limit patient adherence [91]. Notably, propolis is considered safe and well tolerated, particularly when administered orally [21, 92, 93] and it appears to induce fewer adverse effects when compared to chlorhexidine [49]. In the included studies, only two participants reported a slight burning sensation during the first days of propolis use, which resolved spontaneously [49]. However, other adverse effects have been reported in the literature. Propolis may act as a contact sensitizer and can induce allergic reactions in susceptible individuals such as contact allergies and allergic contact dermatitis. The frequency of sensitization has been reported to be approximately 2% in mid- and Eastern European populations [92]. Therefore, caution is warranted when recommending PCFs for patients with a history of allergy to bee products or related substances.

Although this systematic review and meta-analysis suggests that propolis may have a positive effect on caries prevention, most of the evidence relies on surrogate outcomes, including plaque indices and oral microbial counts, which, although relevant, do not directly confirm the preventive effect of PCFs on dental caries. This evidence gap is understandable given the considerable challenges in designing and carrying out medium- or long-term clinical trials capable of verifying any effect on caries incidence. Among the difficulties are patient adherence over extended periods and ethical concerns, since prolonged use of a topical antimicrobial in the oral cavity may disrupt the oral microbiome reducing diversity and shifting the microbial composition, besides potentially fostering antimicrobial resistance [94, 95]. Furthermore, the interpretation of the findings is limited by the methodological quality and inconsistency of the included studies. Most studies presented moderate to high risk of bias, and the certainty of the evidence was graded as very low, reducing confidence in the pooled estimates. In several cases, important methodological details, such as randomization procedures, allocation concealment, blinding, baseline oral health status and treatment protocols were incompletely reported. This may have influenced the magnitude and direction of the observed effects. Notably, for the outcome microbial counts, the statistical significance was no longer maintained after sensitivity analyses excluding studies with a high risk of bias, suggesting that the overall effect for this outcome may have been partially driven by lower-quality evidence.

In conclusion, propolis-containing formulations may reduce plaque accumulation and microbial counts in saliva and oral biofilm, suggesting a possible role in caries prevention. The biofilm reduction achieved with PCFs appears to be similar to that observed with chlorhexidine, which is noteworthy given that propolis may cause fewer side effects. However, well-designed randomized clinical trials assessing direct caries outcomes are needed to properly address the research question. Overall, these results should be interpreted with caution due to high heterogeneity among studies and the very low certainty of the evidence.

## Supplementary Information

Below is the link to the electronic supplementary material.

Supplementary Material 1 (PDF 1.37 MB)

## Data Availability

All data generated or analyzed during this study are included in the article or in the Supplementary Material available on line. For further inquiries, please contact the corresponding author.
