# Supplementary material for: Propolis-containing formulations in caries prevention - a systematic review and meta-analysis
Source: Clin Oral Investig. 2026 Aug 13;30(9):388. doi: 10.1007/s00784-026-07065-6 (PMC13473232; doi:10.1007/s00784-026-07065-6)
Supplement: Supplementary file 1 [file 784_2026_7065_MOESM1_ESM.pdf]

Supplementary material

Clinical Oral Investigations

Propolis-Containing Formulations for Caries Prevention - A Systematic Review and Meta-Analysis

Luciana Pisenti Berto[a]*, Richard Johannes Wierichs[b]*, Letícia Oba Sakae[a], Samira Helena Niemeyer[a], Thiago Saads Carvalho[a]

[a]Department of Restorative, Preventive and Pediatric Dentistry, School of Dental Medicine, University of Bern. [b]Department of Operative Dentistry and Periodontology, University of Bonn. *The authors contributed equally to the study and share first authorship. **Corresponding author:** luciana.pisentiberto@unibe.ch

Table S1 Search strategy used for other databases used in the review, with the number of results.

**CENTRAL* (All text)**		
#1<br>propolis	#2<br>(dental caries) OR (cariogenic) OR (tooth demineralization) OR (tooth remineralization) OR (dental biofilm) OR (dental plaque) OR (plaque index) OR (Streptococcus mutans) OR (antimicrobial)[1]	#1 AND #2
708	38.388	176
**Scopus (TITLE-ABS-KEY)**		
#1<br>(propolis)	#2<br>(caries OR decay OR carious OR cariogenic OR deminerali* OR reminerali* OR "dental biofilm" OR "dental plaque" OR "plaque index" OR "Streptococcus mutans")	#1 AND #2
11.246	686.135	514
**Embase (Broad search)**		
#1<br>propolis	#2<br>'dental caries' OR decay OR cariogenic OR 'tooth demineralization' OR 'dental biofilm' OR 'tooth plaque' OR 'plaque index' OR 'Streptococcus mutans'	#1 AND #2
8.584	221.325	377
**Virtual Health Library (Title, abstract, subject)**		
#1<br>propolis	#2<br>'dental caries' OR decay OR cariogenic OR 'tooth demineralization' OR 'dental biofilm' OR 'tooth plaque' OR 'plaque index' OR 'Streptococcus mutans'	#1 AND #2
6.458	11.605	109
**ClinicalTrials.gov**		
#1 (as "other terms")<br>propolis	#2 (as condition/disease)<br>caries OR decay OR carious OR cariogenic OR demineraliz* OR reminerali* OR "dental biofilm" OR "dental plaque" OR "plaque index" OR "Streptococcus mutans"	#1 AND #2
131	2.239	12
**Google Scholar (all in title)**		
#1<br>propolis	#2<br>'dental OR caries' OR decay OR cariogenic OR 'tooth OR demineralization' OR 'dental OR biofilm' OR 'tooth OR plaque' OR 'plaque OR index' OR 'Streptococcus OR mutans'	#1 AND #2
21.600	1.280.000	338

[1]The term "antimicrobial" was included in this search to increase the number of total hits after "#1 AND #2" search, increasing from 122 hits before inclusion to 151 after inclusion.

Table S2 List of studies excluded after the eligibility assessment, identified by the first author and year of publication. References are provided below.

Study	Reason for exclusion
Gispert Abreu et al 1998 [1]<br>Alkhaled et al 2021 [2]<br>Bogovska-Gigova et al 2025 [3]<br>Darwita et al 2020 [4]<br>Iswari et al 2010 [5]	Not randomized
Andiani et al 2019 **[6]**<br>Darwita et al 2019 **[7]**<br>De-Carli et al 2010 **[8]**<br>Duailibe et al 2007 **[9]**<br>El-Allaky et al 2020 **[10]**<br>El-Allaky et al 2020 **[11]**<br>Ercan et al 2015 **[12]**<br>Finisha et al 2019 **[13]**<br>Gautam et al 2023 **[14]**<br>Hegde et al 2013 **[15]**<br>Khabazian et al 2025 **[16]**<br>Kumar et al 2015 **[17]**<br>Mohsin et al 2015 **[18]**<br>Neto et al 2020 **[19]**<br>Pereira et al 2011 **[20]**<br>Siqueira et al 2021 **[21]**<br>Sukmawati et al 2021 **[22]**<br>Tulsani et al 2014 **[23]**	Did not include a negative control group or chlorhexidine group
Lisbona-Gonzalez et al 2021 **[24]**<br>Sengupta et al 2025 **[25]**	Outcomes not related to dental caries
Chandrashekaraiah et al 2025 **[26]**<br>Mohammed et al 2025 **[27]**	Formulation had other antimicrobial agents
Gispert Abreu et al 2000 **[28]**<br>Naggar et al 2021 **[29]**	Measure of dispersion not reported<br>(Excluded from the meta-analysis)
Anani et al 2023 [30]	Isolated outcome<br>(Excluded from the meta-analysis)

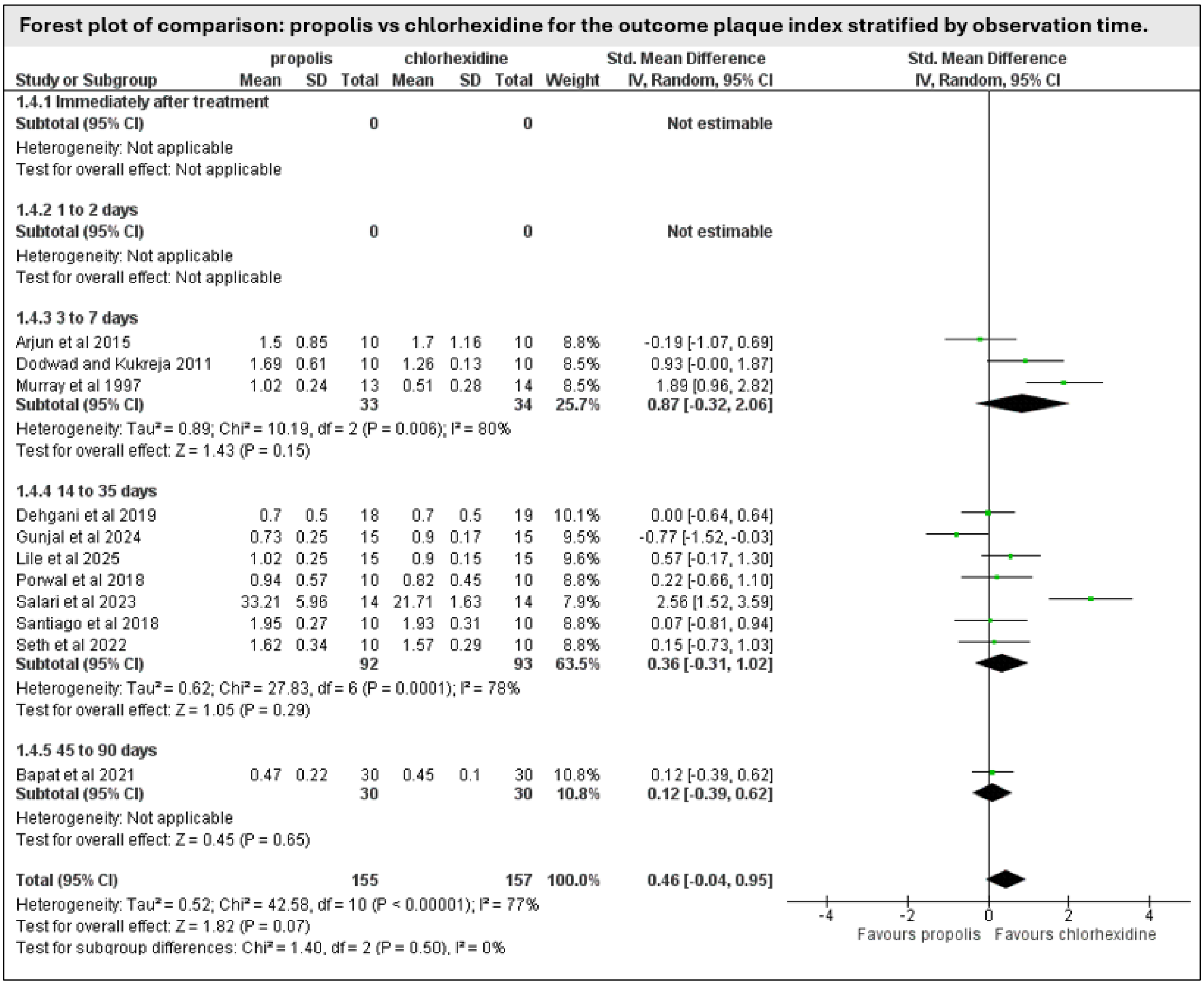

Forest plot of comparison: propolis vs chlorhexidine for the outcome plaque index stratified by observation time.

Study or Subgroup	propolis Mean	propolis SD	propolis Total	chlorhexidine Mean	chlorhexidine SD	chlorhexidine Total	Weight	Std. Mean Difference IV, Random, 95% CI
**1.4.1 Immediately after treatment**								
**Subtotal (95% CI)**			**0**			**0**		**Not estimable**
Heterogeneity: Not applicable								
Test for overall effect: Not applicable								
**1.4.2 1 to 2 days**								
**Subtotal (95% CI)**			**0**			**0**		**Not estimable**
Heterogeneity: Not applicable								
Test for overall effect: Not applicable								
**1.4.3 3 to 7 days**								
Arjun et al 2015	1.5	0.85	10	1.7	1.16	10	8.8%	-0.19 [-1.07, 0.69]
Dodwad and Kukreja 2011	1.69	0.61	10	1.26	0.13	10	8.5%	0.93 [-0.00, 1.87]
Murray et al 1997	1.02	0.24	13	0.51	0.28	14	8.5%	1.89 [0.96, 2.82]
**Subtotal (95% CI)**			**33**			**34**	**25.7%**	**0.87 [-0.32, 2.06]**
Heterogeneity: Tau² = 0.89; Chi² = 10.19, df = 2 (P = 0.006); I² = 80%								
Test for overall effect: Z = 1.43 (P = 0.15)								
**1.4.4 14 to 35 days**								
Dehgani et al 2019	0.7	0.5	18	0.7	0.5	19	10.1%	0.00 [-0.64, 0.64]
Gunjal et al 2024	0.73	0.25	15	0.9	0.17	15	9.5%	-0.77 [-1.52, -0.03]
Lile et al 2025	1.02	0.25	15	0.9	0.15	15	9.6%	0.57 [-0.17, 1.30]
Porwal et al 2018	0.94	0.57	10	0.82	0.45	10	8.8%	0.22 [-0.66, 1.10]
Salari et al 2023	33.21	5.96	14	21.71	1.63	14	7.9%	2.56 [1.52, 3.59]
Santiago et al 2018	1.95	0.27	10	1.93	0.31	10	8.8%	0.07 [-0.81, 0.94]
Seth et al 2022	1.62	0.34	10	1.57	0.29	10	8.8%	0.15 [-0.73, 1.03]
**Subtotal (95% CI)**			**92**			**93**	**63.5%**	**0.36 [-0.31, 1.02]**
Heterogeneity: Tau² = 0.62; Chi² = 27.83, df = 6 (P = 0.0001); I² = 78%								
Test for overall effect: Z = 1.05 (P = 0.29)								
**1.4.5 45 to 90 days**								
Bapat et al 2021	0.47	0.22	30	0.45	0.1	30	10.8%	0.12 [-0.39, 0.62]
**Subtotal (95% CI)**			**30**			**30**	**10.8%**	**0.12 [-0.39, 0.62]**
Heterogeneity: Not applicable								
Test for overall effect: Z = 0.45 (P = 0.65)								
**Total (95% CI)**			**155**			**157**	**100.0%**	**0.46 [-0.04, 0.95]**
Heterogeneity: Tau² = 0.52; Chi² = 42.58, df = 10 (P < 0.00001); I² = 77%								
Test for overall effect: Z = 1.82 (P = 0.07)								
Test for subgroup differences: Chi² = 1.40, df = 2 (P = 0.50), I² = 0%								

Fig. S1 Subgroup analysis by observation time for the outcome plaque index comparing propolis versus chlorhexidine

Forest plot of comparison: propolis vs control for the outcome plaque index stratified by observation time.

Study or Subgroup	propolis Mean	SD	Total	negative control Mean	SD	Total	Weight	Std. Mean Difference IV, Random, 95% CI
**1.2.1 Immediately after treatment**								
**Subtotal (95% CI)**			**0**			**0**		**Not estimable**
Heterogeneity: Not applicable								
Test for overall effect: Not applicable								
**1.2.2 1 to 2 days**								
Bhat et al 2015	35.88	2.36	30	37.48	2.25	30	4.5%	-0.68 [-1.21, -0.16]
Raman and Jain 2023	38.72	2.4	80	45.4	5.08	80	4.7%	-1.67 [-2.03, -1.31]
Ranjan and Anshuman 2023	36.74	2.4	50	46.44	5.07	50	4.5%	-2.43 [-2.95, -1.91]
**Subtotal (95% CI)**			**160**			**160**	**13.8%**	**-1.60 [-2.48, -0.72]**
Heterogeneity: Tau² = 0.55; Chi² = 21.64, df = 2 (P < 0.0001); I² = 91%								
Test for overall effect: Z = 3.55 (P = 0.0004)								
**1.2.3 3 to 7 days**								
Arjun et al 2015	1.5	0.85	10	2.9	0.87	10	3.8%	-1.56 [-2.59, -0.53]
Dodwad and Kukreja 2011	1.69	0.61	10	1.96	1.22	10	4.0%	-0.27 [-1.15, 0.61]
Hassan et al 2021	0.69	0.4	20	0.89	0.5	20	4.4%	-0.43 [-1.06, 0.19]
Koo et al 2002	0.78	0.17	6	1.41	0.14	6	2.1%	-3.73 [-5.88, -1.59]
Murray et al 1997	1.02	0.24	13	1.18	0.36	14	4.2%	-0.50 [-1.27, 0.27]
**Subtotal (95% CI)**			**59**			**60**	**18.5%**	**-0.92 [-1.65, -0.18]**
Heterogeneity: Tau² = 0.44; Chi² = 12.22, df = 4 (P = 0.02); I² = 67%								
Test for overall effect: Z = 2.45 (P = 0.01)								
**1.2.4 14 to 35 days**								
Amano et al 2024	0.225	0.327	45	0.245	0.163	45	4.7%	-0.08 [-0.49, 0.34]
Biria er al 2019	2.342	0.90929	30	3.9067	1.50151	30	4.5%	-1.24 [-1.80, -0.69]
Gunjal et al 2024	0.73	0.25	15	1.24	0.24	15	4.0%	-2.02 [-2.93, -1.12]
Lile et al 2025	1.02	0.25	15	2.01	0.3	15	3.5%	-3.49 [-4.68, -2.30]
Lotif et al 2022	20.6	16.44	21	27.4	14.63	21	4.4%	-0.43 [-1.04, 0.18]
Machorowska-Pienidhek 2013	62	28.43	21	63.97	26.44	20	4.4%	-0.07 [-0.68, 0.54]
Machorowska-Pienidhek et al 2016	1.25	1	37	0.96	0.42	37	4.6%	0.37 [-0.09, 0.83]
Niedzielska et al 2016	31.85	14.89	16	36.98	12.36	15	4.3%	-0.36 [-1.08, 0.35]
Santiago et al 2018	1.95	0.27	10	2.88	0.42	10	3.4%	-2.52 [-3.76, -1.29]
Silva et al 2024	20.6	16.44	38	27.4	14.63	38	4.6%	-0.43 [-0.89, 0.02]
**Subtotal (95% CI)**			**248**			**246**	**42.4%**	**-0.89 [-1.44, -0.33]**
Heterogeneity: Tau² = 0.67; Chi² = 72.46, df = 9 (P < 0.00001); I² = 88%								
Test for overall effect: Z = 3.12 (P = 0.002)								
**1.2.5 45 to 90 days**								
Aggarwal et al 2023	1.77	0.39	10	1.69	0.5	10	4.0%	0.17 [-0.71, 1.05]
Bakery et al 2023	0.683	0.05	40	0.795	0.022	20	4.3%	-2.58 [-3.30, -1.86]
Bapat et al 2021	0.47	0.22	30	1.04	0.08	30	4.1%	-3.40 [-4.21, -2.59]
Pundir et al 2017	1.28	0.1	15	1.56	0.4	15	4.2%	-0.93 [-1.69, -0.18]
Sahu et al 2023	0.715	0.244	20	0.82	0.122	20	4.4%	-0.53 [-1.17, 0.10]
Tanasiewicz et al 2012	21.6	16.8	20	24.9	20.9	20	4.4%	-0.17 [-0.79, 0.45]
**Subtotal (95% CI)**			**135**			**115**	**25.4%**	**-1.24 [-2.32, -0.15]**
Heterogeneity: Tau² = 1.70; Chi² = 67.18, df = 5 (P < 0.00001); I² = 93%								
Test for overall effect: Z = 2.24 (P = 0.03)								
**Total (95% CI)**			**602**			**581**	**100.0%**	**-1.11 [-1.52, -0.70]**
Heterogeneity: Tau² = 0.92; Chi² = 227.32, df = 23 (P < 0.00001); I² = 90%								
Test for overall effect: Z = 5.25 (P < 0.00001)								
Test for subgroup differences: Chi² = 2.04, df = 3 (P = 0.56), I² = 0%								

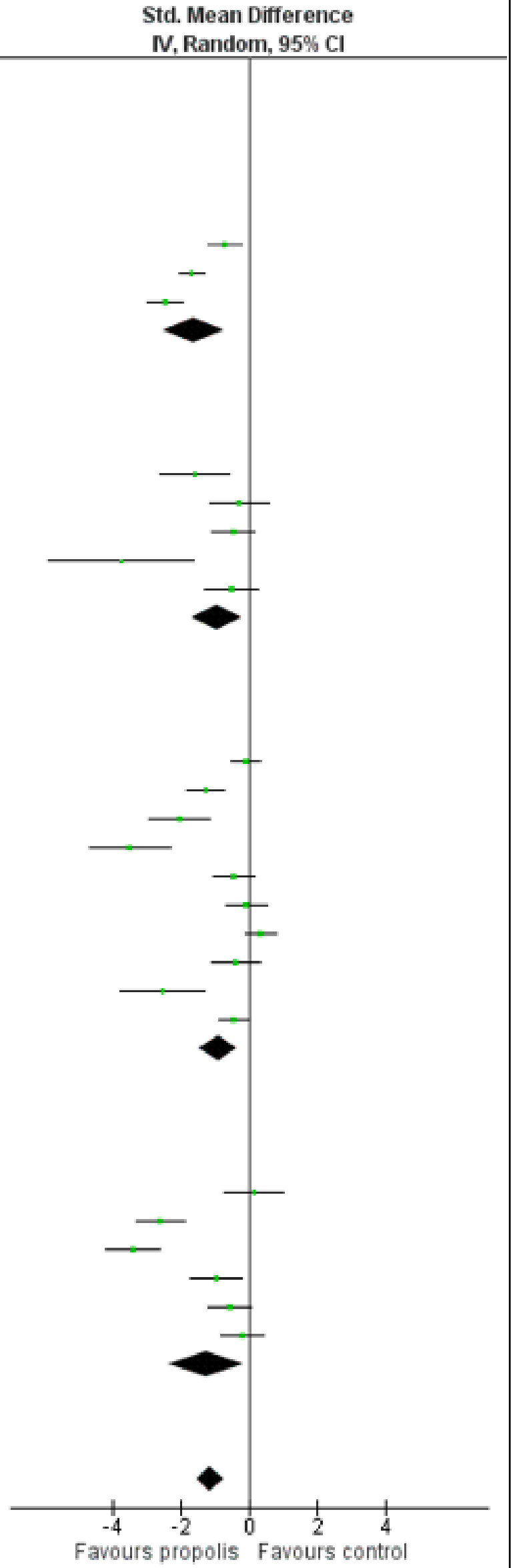

Fig. S2 Subgroup analysis by observation time for the outcome plaque index comparing propolis versus negative control

Forest plot of comparison: propolis vs control for the outcome microbial counts stratified by observation time.

Study or Subgroup	propolis Mean	propolis SD	propolis Total	negative control Mean	negative control SD	negative control Total	Weight	Std. Mean Difference IV, Random, 95% CI
**1.1.1 Immediately after treatment**								
Prabhakar et al 2015 (total microorganisms)	4.5	5.23	10	47.2	26.61	10	8.7%	-2.13 [-3.28, -0.99]
Uday Mohan et al 2016 (S. mutans) Δ	-42.8	35.7	20	-114	63.4	20	9.4%	1.36 [0.66, 2.05]
**Subtotal (95% CI)**			**30**			**30**	**18.2%**	**-0.36 [-3.78, 3.06]**
Heterogeneity: Tau² = 5.85; Chi² = 26.06, df = 1 (P < 0.00001); I² = 96%								
Test for overall effect: Z = 0.20 (P = 0.84)								
**1.1.3 1 to 7 days**								
Lisbona-Gonzales et al 2021a (S. mutans)	1.4	0.516	10	2	0.00001	10	8.9%	-1.57 [-2.61, -0.54]
Martins et al 2020 (Streptococcus spp.) Δ	0.714	1.459	29	-0.56	1.527	29	9.6%	0.84 [0.30, 1.38]
Soekanto et al 2018 (S. mutans)	6.832	0.307	6	7.945	0.394	6	7.4%	-2.91 [-4.72, -1.09]
**Subtotal (95% CI)**			**45**			**45**	**25.9%**	**-1.10 [-3.31, 1.12]**
Heterogeneity: Tau² = 3.47; Chi² = 27.95, df = 2 (P < 0.00001); I² = 93%								
Test for overall effect: Z = 0.97 (P = 0.33)								
**1.1.4 14 to 35 days**								
Furtado Junior et al 2020 (S. mutans)	2.66	0.49	46	3.5	0.47	46	9.7%	-1.73 [-2.22, -1.25]
Lotif et al 2022 (Lactobacilli) Δ	-0.56	0.34	21	0.59	0.36	21	9.1%	-3.22 [-4.17, -2.28]
**Subtotal (95% CI)**			**67**			**67**	**18.8%**	**-2.42 [-3.87, -0.97]**
Heterogeneity: Tau² = 0.96; Chi² = 7.57, df = 1 (P = 0.006); I² = 87%								
Test for overall effect: Z = 3.27 (P = 0.001)								
**1.1.5 45 to 90 days**								
Anani et al 2023 (S. mutans)	89.83	6.14	8	89.61	7.8	8	9.0%	0.03 [-0.95, 1.01]
Anauete Netto et al 2013 (S. mutans)	1.4	0.5	20	2.6	0.7	20	9.3%	-1.93 [-2.70, -1.17]
Bakery et al 2023 (S. mutans)	446	4.961	40	470	8.87	20	9.2%	-3.64 [-4.50, -2.78]
Bapat et al 2021 (S. mutans)	610	453.606	30	48,000	43,151.69	30	9.6%	-1.53 [-2.11, -0.95]
**Subtotal (95% CI)**			**98**			**78**	**37.1%**	**-1.78 [-3.05, -0.51]**
Heterogeneity: Tau² = 1.50; Chi² = 31.86, df = 3 (P < 0.00001); I² = 91%								
Test for overall effect: Z = 2.76 (P = 0.006)								
**Total (95% CI)**			**240**			**220**	**100.0%**	**-1.45 [-2.43, -0.48]**
Heterogeneity: Tau² = 2.50; Chi² = 177.08, df = 10 (P < 0.00001); I² = 94%								
Test for overall effect: Z = 2.92 (P = 0.004)								
Test for subgroup differences: Chi² = 1.77, df = 3 (P = 0.62), I² = 0%								

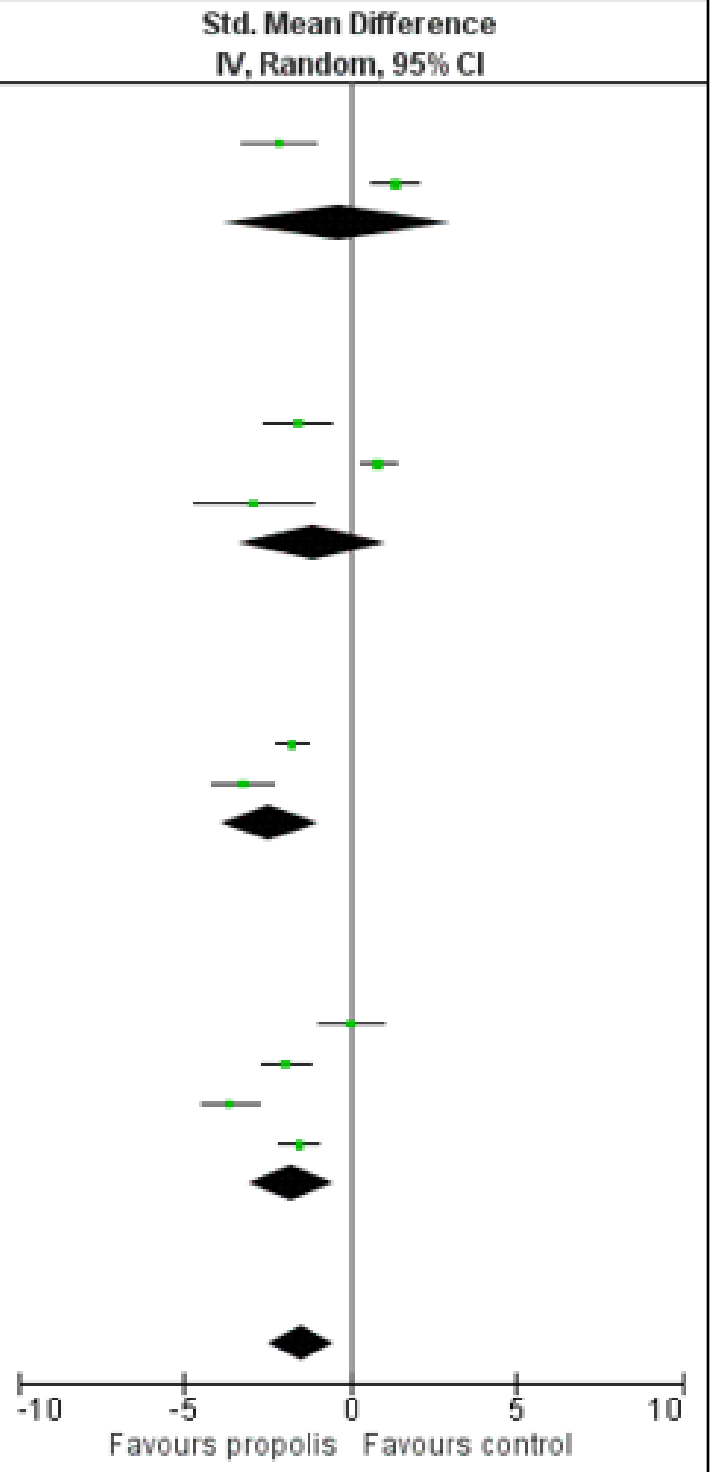

Fig. S3 Subgroup analysis by observation time for the outcome microbial counts comparing propolis versus negative control

Forest plot of comparison: propolis vs chlorhexidine for the outcome microbial counts stratified by observation time.

Study or Subgroup	propolis Mean	propolis SD	propolis Total	chlorhexidine Mean	chlorhexidine SD	chlorhexidine Total	Weight	Std. Mean Difference IV, Random, 95% CI
**1.3.1 Immediately after treatment**								
Alkhaled et al 2021 (S. mutans)	15.9	4.48	20	25.2	4.94	20	12.0%	-1.93 [-2.70, -1.17]
Guru et al 2019 (S. mutans)	21.67	21.019	60	7.62	13.357	60	13.5%	0.79 [0.42, 1.16]
Uday Mohan et al 2016 (S. mutans) Δ	-42.8	35.7	20	-114	63.4	20	12.3%	1.36 [0.66, 2.05]
**Subtotal (95% CI)**			**100**			**100**	**37.8%**	**0.09 [-1.59, 1.77]**
Heterogeneity: Tau² = 2.09; Chi² = 47.19, df = 2 (P < 0.00001); I² = 96%								
Test for overall effect: Z = 0.10 (P = 0.92)								
**1.3.2 1 to 2 days**								
Lisbona-Gonzales et al 2021a (S. mutans)	1.4	0.516	10	1.2	0.421	10	11.4%	0.41 [-0.48, 1.29]
**Subtotal (95% CI)**			**10**			**10**	**11.4%**	**0.41 [-0.48, 1.29]**
Heterogeneity: Not applicable								
Test for overall effect: Z = 0.90 (P = 0.37)								
**1.3.3 3 to 7 days**								
**Subtotal (95% CI)**			**0**			**0**		**Not estimable**
Heterogeneity: Not applicable								
Test for overall effect: Not applicable								
**1.3.4 14 to 35 days**								
Seth et al 2022 (total microorganisms)	82.5	13.59	20	75	14.28	20	12.6%	0.53 [-0.10, 1.16]
**Subtotal (95% CI)**			**20**			**20**	**12.6%**	**0.53 [-0.10, 1.16]**
Heterogeneity: Not applicable								
Test for overall effect: Z = 1.64 (P = 0.10)								
**1.3.5 45 to 90 days**								
Anauete Netto et al 2013 (S. mutans)	1.4	0.5	20	1.9	0.5	20	12.5%	-0.98 [-1.64, -0.32]
Bapat et al 2021 (S. mutans)	610	453.6	30	580	456.67	30	13.1%	0.07 [-0.44, 0.57]
Neto et al 2021(S. mutans)	0.64	0.36	25	0.31	0.18	25	12.7%	1.14 [0.54, 1.74]
**Subtotal (95% CI)**			**75**			**75**	**38.2%**	**0.08 [-1.04, 1.20]**
Heterogeneity: Tau² = 0.89; Chi² = 21.80, df = 2 (P < 0.0001); I² = 91%								
Test for overall effect: Z = 0.14 (P = 0.89)								
**Total (95% CI)**			**205**			**205**	**100.0%**	**0.19 [-0.49, 0.87]**
Heterogeneity: Tau² = 0.85; Chi² = 71.64, df = 7 (P < 0.00001); I² = 90%								
Test for overall effect: Z = 0.54 (P = 0.59)								
Test for subgroup differences: Chi² = 0.60, df = 3 (P = 0.90), I² = 0%								

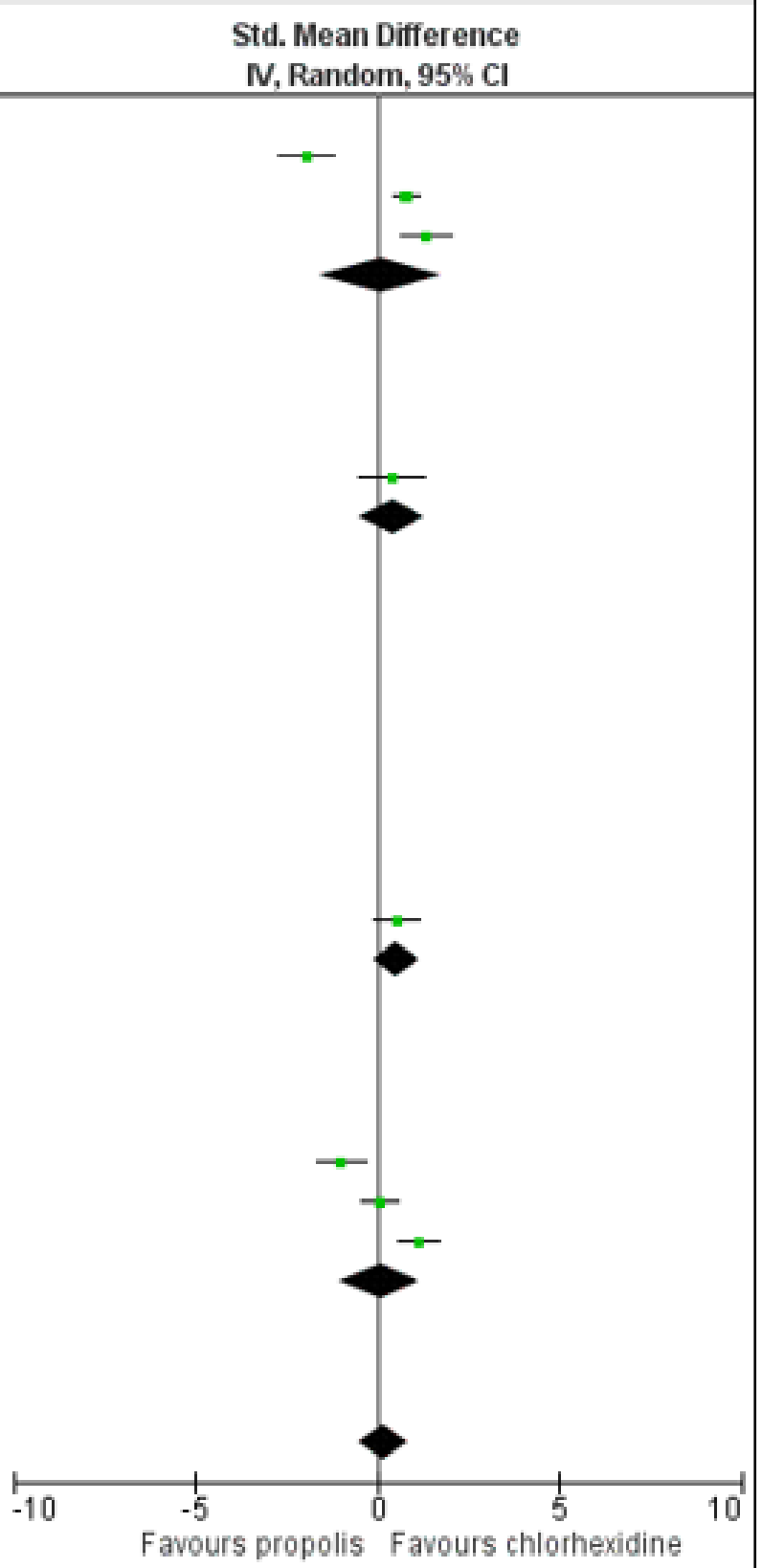

Fig. S4 Subgroup analysis by observation time for the outcome microbial counts comparing propolis versus chlorhexidine

Forest plot of comparison: propolis vs control for the outcome plaque index stratified by type of formulation.

Study or Subgroup	propolis Mean	propolis SD	propolis Total	negative control Mean	negative control SD	negative control Total	Weight	Std. Mean Difference IV, Random, 95% CI
**1.2.1 Mouthwash**								
Arjun et al 2015	1.5	0.85	10	2.9	0.87	10	7.9%	-1.56 [-2.59, -0.53]
Bakery et al 2023	0.683	0.05	40	0.795	0.022	20		Not estimable
Bapat et al 2021	0.47	0.22	30	1.04	0.08	30		Not estimable
Dodwad and Kukreja 2011	1.69	0.61	10	1.96	1.22	10		Not estimable
Gunjal et al 2024	0.73	0.25	15	1.24	0.24	15	8.5%	-2.02 [-2.93, -1.12]
Koo et al 2002	0.78	0.17	6	1.41	0.14	6	3.8%	-3.73 [-5.88, -1.59]
Lile et al 2025	1.02	0.25	15	2.01	0.3	15	7.1%	-3.49 [-4.68, -2.30]
Murray et al 1997	1.02	0.24	13	1.18	0.36	14		Not estimable
Pundir et al 2017	1.28	0.1	15	1.56	0.4	15		Not estimable
Santiago et al 2018	1.95	0.27	10	2.88	0.42	10		Not estimable
**Subtotal (95% CI)**			**46**			**46**	**27.3%**	**-2.51 [-3.49, -1.53]**
Heterogeneity: Tau² = 0.59; Chi² = 7.90, df = 3 (P = 0.05); I² = 62%								
Test for overall effect: Z = 5.02 (P < 0.00001)								
**1.2.2 Dentifrice**								
Amano et al 2024	0.225	0.327	45	0.245	0.163	45	10.8%	-0.08 [-0.49, 0.34]
Bhat et al 2015	35.88	2.36	30	37.48	2.25	30	10.3%	-0.68 [-1.21, -0.16]
Biria er al 2019	2.342	0.90929	30	3.9067	1.50151	30	10.2%	-1.24 [-1.80, -0.69]
Hassan et al 2021	0.69	0.4	20	0.89	0.5	20		Not estimable
Lotif et al 2022	20.6	16.44	21	27.4	14.63	21	9.9%	-0.43 [-1.04, 0.18]
Machorowska-Pienidhek 2013	62	28.43	21	63.97	26.44	20		Not estimable
Machorowska-Pienidhek et al 2016	1.25	1	37	0.96	0.42	37		Not estimable
Niedzielska et al 2016	31.85	14.89	16	36.98	12.36	15		Not estimable
Raman and Jain 2023	38.72	2.4	80	45.4	5.08	80	11.0%	-1.67 [-2.03, -1.31]
Ranjan and Anshuman 2023	36.74	2.4	50	46.44	5.07	50		Not estimable
Silva et al 2024	20.6	16.44	38	27.4	14.63	38	10.6%	-0.43 [-0.89, 0.02]
Tanasiewicz et al 2012	21.6	16.8	20	24.9	20.9	20		Not estimable
**Subtotal (95% CI)**			**244**			**244**	**62.8%**	**-0.76 [-1.31, -0.21]**
Heterogeneity: Tau² = 0.41; Chi² = 40.70, df = 5 (P < 0.00001); I² = 88%								
Test for overall effect: Z = 2.71 (P = 0.007)								
**1.2.3 Gel or solution for subgingival application**								
Aggarwal et al 2023	1.77	0.39	10	1.69	0.5	10		Not estimable
Sahu et al 2023	0.715	0.244	20	0.82	0.122	20	9.8%	-0.53 [-1.17, 0.10]
**Subtotal (95% CI)**			**20**			**20**	**9.8%**	**-0.53 [-1.17, 0.10]**
Heterogeneity: Not applicable								
Test for overall effect: Z = 1.65 (P = 0.10)								
**Total (95% CI)**			**310**			**310**	**100.0%**	**-1.22 [-1.73, -0.71]**
Heterogeneity: Tau² = 0.59; Chi² = 74.86, df = 10 (P < 0.00001); I² = 87%								
Test for overall effect: Z = 4.67 (P < 0.00001)								
Test for subgroup differences: Chi² = 11.84, df = 2 (P = 0.003), I² = 83.1%								

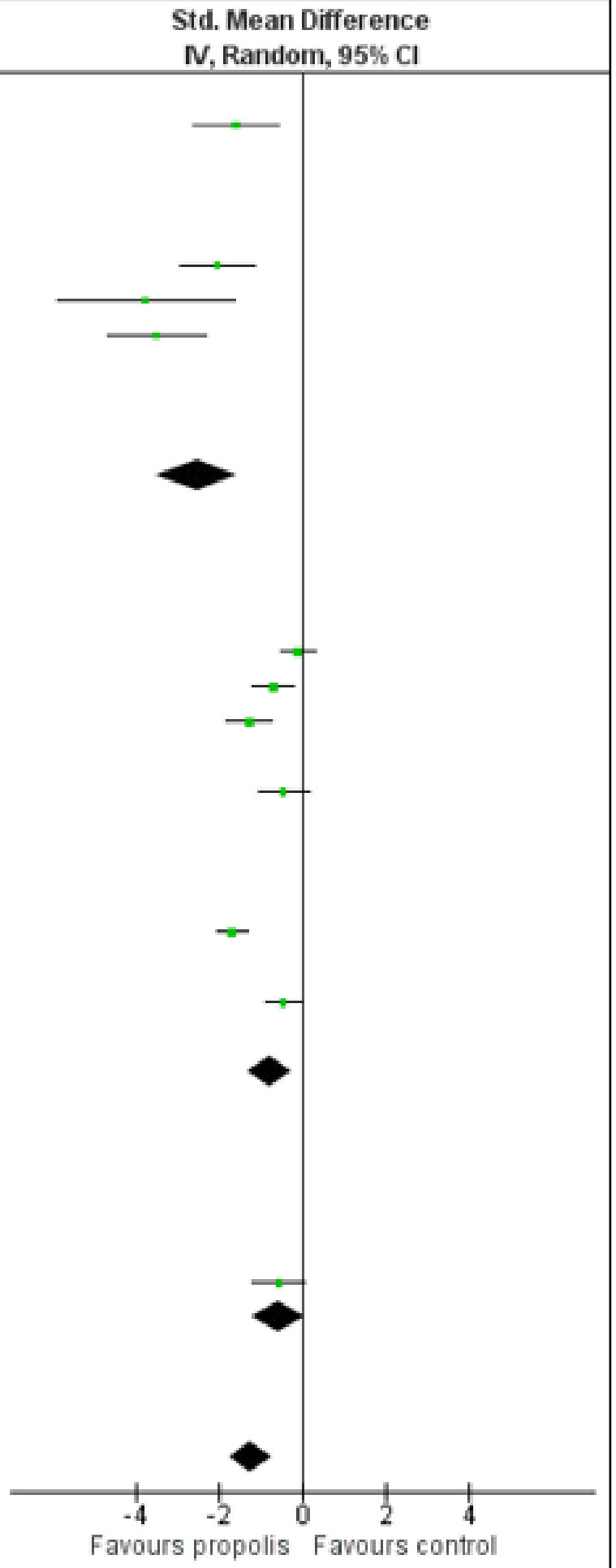

Fig. S5 Sensitivity analysis for the outcome plaque index comparing propolis versus negative control, excluding high-risk-of-bias studies. "Δ" indicates that the study reported results as the difference between baseline and post-treatment; these values were multiplied by –1 to align the direction of effect with the other studies

Forest plot of comparison: propolis vs chlorhexidine for the outcome plaque index.

Study or Subgroup	propolis Mean	propolis SD	propolis Total	chlorhexidine Mean	chlorhexidine SD	chlorhexidine Total	Weight	Std. Mean Difference IV, Random, 95% CI
Arjun et al 2015	1.5	0.85	10	1.7	1.16	10	16.9%	-0.19 [-1.07, 0.69]
Bapat et al 2021	0.47	0.22	30	0.45	0.1	30		Not estimable
Dehgani et al 2019	0.7	0.5	18	0.7	0.5	19	24.3%	0.00 [-0.64, 0.64]
Dodwad and Kukreja 2011	1.69	0.61	10	1.26	0.13	10		Not estimable
Gunjal et al 2024	0.73	0.25	15	0.9	0.17	15	20.7%	-0.77 [-1.52, -0.03]
Lile et al 2025	1.02	0.25	15	0.9	0.15	15	21.2%	0.57 [-0.17, 1.30]
Murray et al 1997	1.02	0.24	13	0.51	0.28	14		Not estimable
Porwal et al 2018	0.94	0.57	10	0.82	0.45	10		Not estimable
Salari et al 2023	33.21	5.96	14	21.71	1.63	14		Not estimable
Santiago et al 2018	1.95	0.27	10	1.93	0.31	10		Not estimable
Seth et al 2022	1.62	0.34	10	1.57	0.29	10	16.9%	0.15 [-0.73, 1.03]
**Total (95% CI)**			**68**			**69**	**100.0%**	**-0.05 [-0.49, 0.40]**
Heterogeneity: Tau² = 0.10; Chi² = 6.66, df = 4 (P = 0.15); I² = 40%								
Test for overall effect: Z = 0.21 (P = 0.84)								

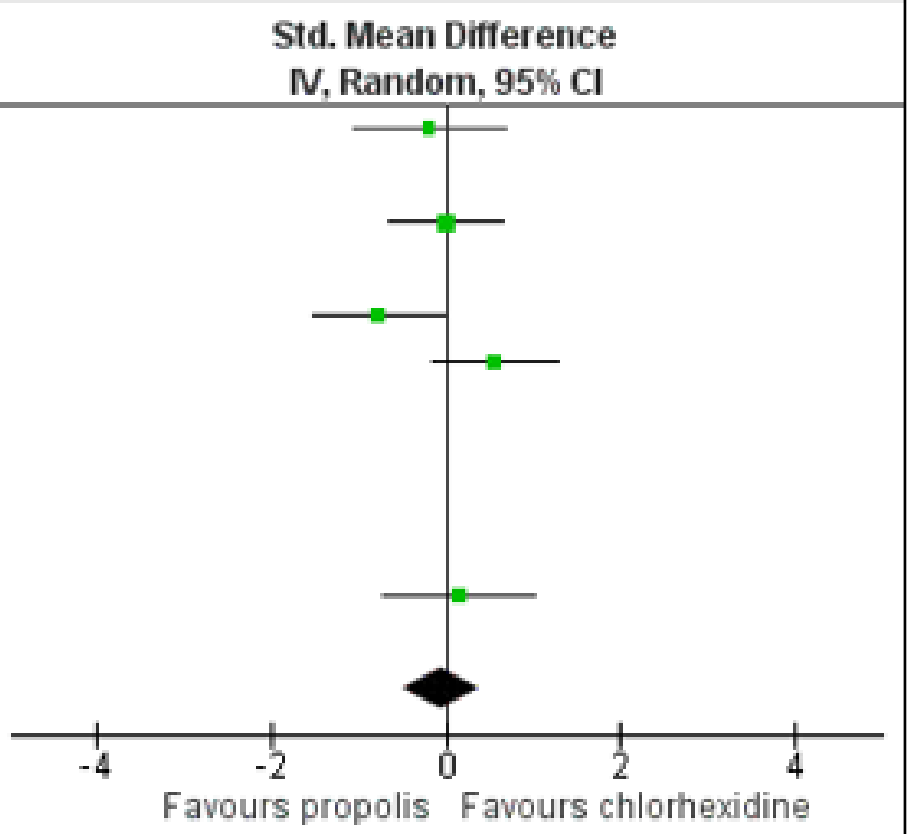

Fig. S6 Sensitivity analysis for the outcome plaque index comparing propolis versus chlorhexidine, excluding high-risk-of-bias studies

Forest plot of comparison: propolis vs control for the outcome microbial counts stratified by type of formulation.

Study or Subgroup	propolis Mean	propolis SD	propolis Total	negative control Mean	negative control SD	negative control Total	Weight	Std. Mean Difference IV, Random, 95% CI
**1.1.1 Mouthwashes**								
Anauete Netto et al 2013 (S. mutans)	1.4	0.5	20	2.6	0.7	20	16.7%	-1.93 [-2.70, -1.17]
Bakery et al 2023 (S. mutans)	446	4.961	40	470	8.87	20		Not estimable
Bapat et al 2021 (S. mutans)	610	453.606	30	48,000	43,151.69	30		Not estimable
Lisbona-Gonzales et al 2021a (S. mutans)	1.4	0.516	10	2	0.00001	10	16.0%	-1.57 [-2.61, -0.54]
**Subtotal (95% CI)**			**30**			**30**	**32.7%**	**-1.81 [-2.42, -1.19]**
Heterogeneity: Tau² = 0.00; Chi² = 0.30, df = 1 (P = 0.58); I² = 0%								
Test for overall effect: Z = 5.77 (P < 0.00001)								
**1.1.2 Dentifrices**								
Furtado Junior et al 2020 (S. mutans)	2.66	0.49	46	3.5	0.47	46	17.2%	-1.73 [-2.22, -1.25]
Lotif et al 2022 (Lactobacilli) Δ	-0.56	0.34	21	0.59	0.36	21	16.2%	-3.22 [-4.17, -2.28]
**Subtotal (95% CI)**			**67**			**67**	**33.4%**	**-2.42 [-3.87, -0.97]**
Heterogeneity: Tau² = 0.96; Chi² = 7.57, df = 1 (P = 0.006); I² = 87%								
Test for overall effect: Z = 3.27 (P = 0.001)								
**1.1.3 Application on carious dentine**								
Anani et al 2023 (S. mutans)	89.83	6.14	8	89.61	7.8	8		Not estimable
Prabhakar et al 2015 (total microorganisms)	4.5	5.23	10	47.2	26.61	10		Not estimable
Uday Mohan et al 2016 (S. mutans) Δ	-42.8	35.7	20	-114	63.4	20	16.8%	1.36 [0.66, 2.05]
**Subtotal (95% CI)**			**20**			**20**	**16.8%**	**1.36 [0.66, 2.05]**
Heterogeneity: Not applicable								
Test for overall effect: Z = 3.83 (P = 0.0001)								
**1.1.4 Tablet or candy**								
Martins et al 2020 (Streptococcus spp.) Δ	0.714	1.459	29	-0.56	1.527	29	17.1%	0.84 [0.30, 1.38]
Soekanto et al 2018 (S. mutans)	6.832	0.307	6	7.945	0.394	6		Not estimable
**Subtotal (95% CI)**			**29**			**29**	**17.1%**	**0.84 [0.30, 1.38]**
Heterogeneity: Not applicable								
Test for overall effect: Z = 3.06 (P = 0.002)								
**Total (95% CI)**			**146**			**146**	**100.0%**	**-1.02 [-2.42, 0.37]**
Heterogeneity: Tau² = 2.88; Chi² = 122.98, df = 5 (P < 0.00001); I² = 96%								
Test for overall effect: Z = 1.44 (P = 0.15)								
Test for subgroup differences: Chi² = 67.95, df = 3 (P < 0.00001), I² = 95.6%								

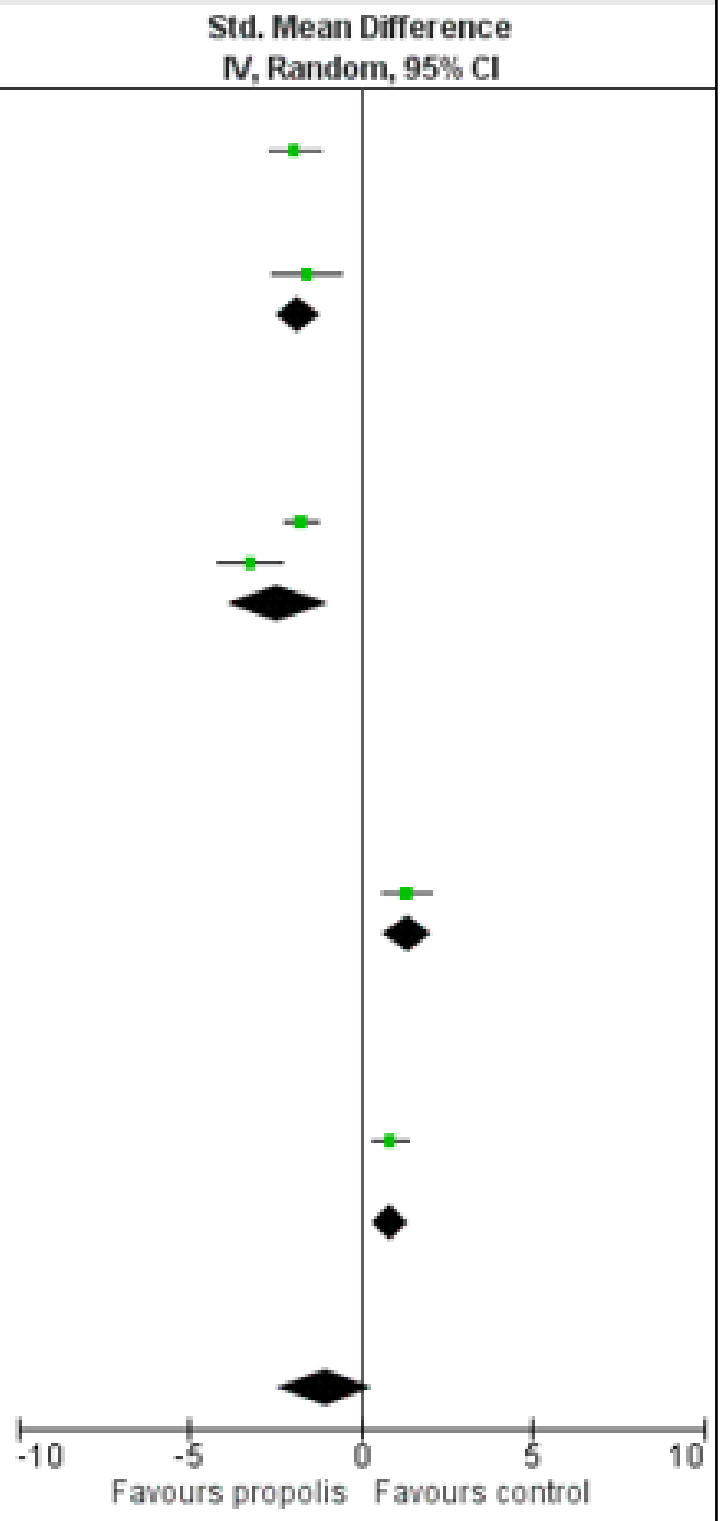

Fig. S7 Sensitivity analysis for the outcome microbial counts comparing propolis versus negative control, excluding high-risk-of-bias studies. "Δ" indicates that the study reported results as the difference between baseline and post-treatment; these values were multiplied by –1 to align the direction of effect with the other studies

Forest plot of comparison: propolis vs chlorhexidine for the outcome microbial counts.

Study or Subgroup	propolis Mean	propolis SD	propolis Total	chlorhexidine Mean	chlorhexidine SD	chlorhexidine Total	Weight	Std. Mean Difference IV, Random, 95% CI
Anauete Netto et al 2013 (S. mutans)	1.4	0.5	20	1.9	0.5	20	20.3%	-0.98 [-1.64, -0.32]
Bapat et al 2021 (S. mutans)	610	453.6	30	580	456.67	30		Not estimable
Guru et al 2019 (S. mutans)	21.67	21.019	60	7.62	13.357	60		Not estimable
Lisbona-Gonzales et al 2021a (S. mutans)	1.4	0.516	10	1.2	0.421	10	18.4%	0.41 [-0.48, 1.29]
Neto et al 2021(S. mutans)	0.64	0.36	25	0.31	0.18	25	20.8%	1.14 [0.54, 1.74]
Seth et al 2022 (total microorganisms)	82.5	13.59	20	75	14.28	20	20.5%	0.53 [-0.10, 1.16]
Uday Mohan et al 2016 (S. mutans) Δ	-42.8	35.7	20	-114	63.4	20	20.0%	1.36 [0.66, 2.05]
**Total (95% CI)**			**95**			**95**	**100.0%**	**0.49 [-0.34, 1.32]**
Heterogeneity: Tau² = 0.77; Chi² = 29.59, df = 4 (P < 0.00001); I² = 86%								
Test for overall effect: Z = 1.16 (P = 0.25)								

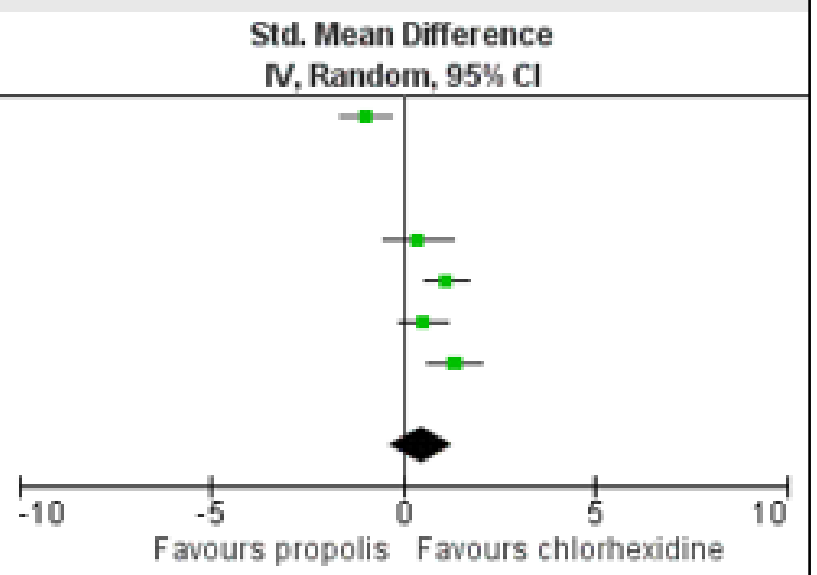

Fig. S8 Sensitivity analysis for the outcome microbial counts comparing propolis versus chlorhexidine, excluding high-risk-of-bias studies. "Δ" indicates that the study reported results as the difference between baseline and post-treatment; these values were multiplied by –1 to align the direction of effect with the other studies

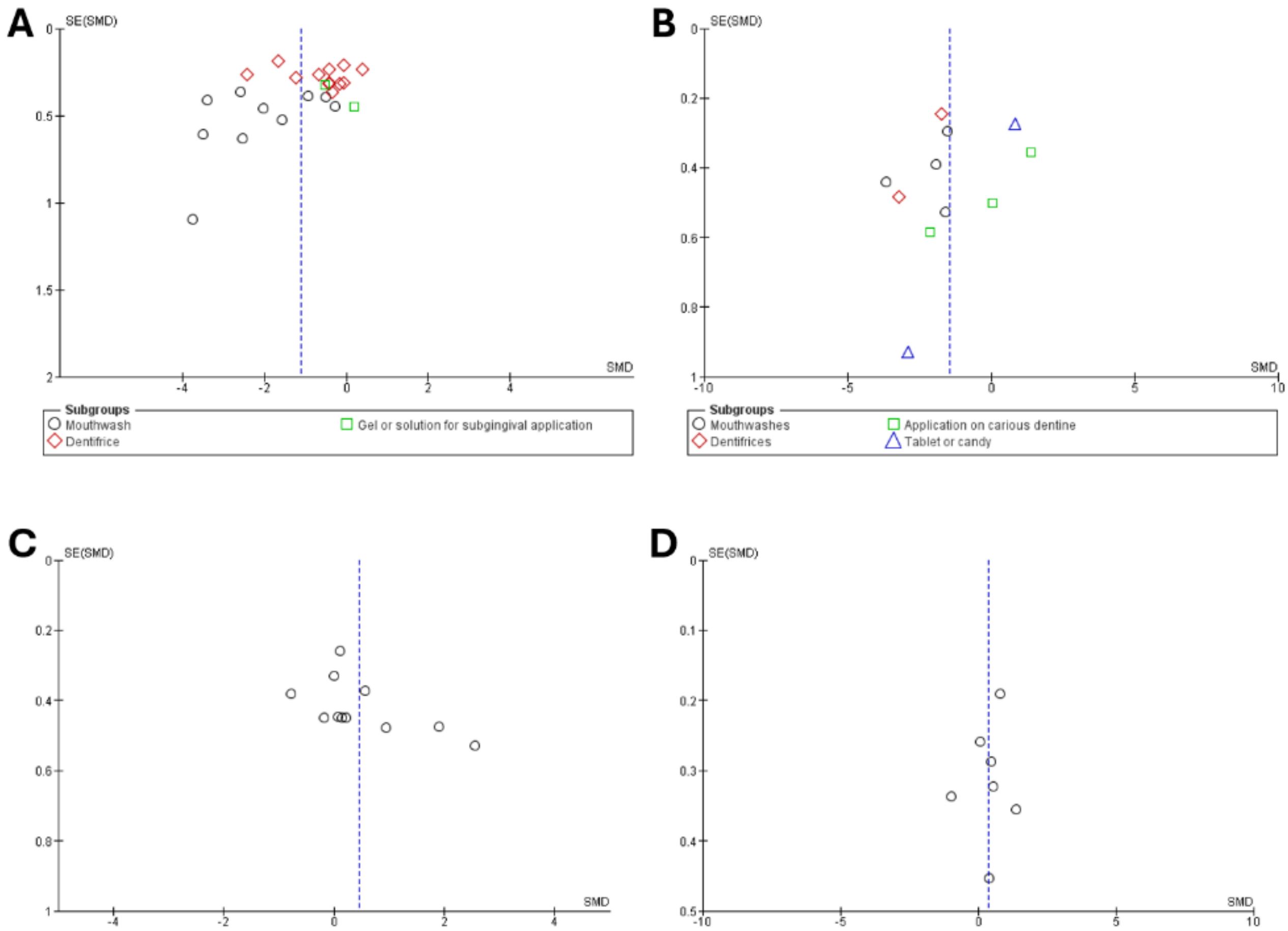

Fig. S9 Funnel plots of studies included in the meta-analysis assessing publication bias. Plots are shown for plaque index comparing propolis-containing formulations with a negative control (A) and chlorhexidine (C), and for microbial counts comparing propolis-containing formulations with a negative control (B) and chlorhexidine (D)

Table S3 Pooled effect estimates and heterogeneity for plaque index and microbial counts when including or excluding studies primarily investigating gingivitis or periodontitis.

Outcome	Control	Including/ excluding studies with periodontal patients	SMD [95% CI]	p value	I^2
Plaque index	Placebo	Including	-1.11 [-1.52, -0.70]	<0.001	90%
		Excluding	-1.26 [-1.79, -0.73]	<0.001	92%
	Chlorhexidine	Including	0.46 [-0.04, 0.95]	0.070	77%
		Excluding	0.47 [-0.11, 1.05]	0.110	70%
Microbial counts	Placebo	Including	-1.45 [-2.43, -0.48]	0.004	94%
		Excluding	-1.44 [-2.49, -0.39]	0.007	95%
	Chlorhexidine	Including	0.38 [-0.11, 0.87]	0.130	80%
		Excluding	0.34 [-0.32, 1.00]	0.310	87%

Table S4 GRADE evidence profile of included studies.

Certainty assessment							Summary of findings			Certainty	Importance
№ of studies	Study design	Risk of bias	Inconsistency	Indirectness	Imprecision	Other considerations	№ of patients		Effect		
							Propolis	Control	Absolute (95% CI)		
**Plaque index (propolis vs. negative control)**											
26	randomised trials	serious [a]	serious [b]	serious [c]	not serious [d]	none	602	581	SMD **1.11 SD lower** (1.52 lower to 0.70 lower)	◯◯◯⊗ very low	IMPORTANT
**Plaque index (propolis vs. chlorhexidine)**											
11	randomised trials	serious [a]	serious [b]	serious [c]	not serious [d]	none	155	157	SMD **0.46 SD higher** (0.04 lower to 0.95 higher)	◯◯◯⊗ very low	IMPORTANT
**Microbial counts (propolis vs. negative control)**											
12	randomised trials	serious [a]	serious [b]	serious [c]	not serious [d]	none	240	220	SMD **1.45 SD lower** (2.43 lower to 0.48 lower)	◯◯◯⊗ very low	IMPORTANT
**Microbial counts (propolis vs. chlorhexidine)**											
8	randomised trials	serious [a]	serious [b]	serious [c]	not serious [d]	none	185	185	SMD **0.38 SD higher** (0.11 lower to 0.87 higher)	◯◯◯⊗ very low	IMPORTANT

[a] Studies lack clear information about randomization, blinding, and selection of outcomes. Most of the included studies did not register the trial protocol in a public database (see risk of bias analysis – Fig. 3 in the main document).

[b] Studies show considerable heterogeneity (I^2>90% - see forest plots and meta-analyses in the main document).

[c] The research question is not directly answered by the analysis of this outcome alone. Direct caries outcomes such as caries indices were rarely found and most studies reported surrogate outcomes related to caries risk such as plaque index and saliva or biofilm microbial count.

[d] Confidence intervals were overall narrow (see forest plots in the main document) and the number of total evaluated patients for the outcome was higher than the Optimal Information Size (128 - calculated considering α=0.05, β=0.2 and effect size=0.5).

Supplementary references

1. Gispert Abreu E, Cantillo Estrada E, Rivero López A, Oramas Rodríguez B (1998) Estudio comparativo del efecto del cepillado con una crema dental con propóleos rojos y de un gel con clorofila. Revista Cubana de Estomatología 35:112-118.
2. Alkhaled A, Alsabek L, Al-assaf M, Badr F (2021) Effect of chlorhexidine, honey and propolis on *Streptococcus mutans* counts: in vitro study. Dentistry 3000 9. https://doi.org/10.5195/d3000.2021.166.
3. Bogovska-Gigova R, Hristov K (2025) Effect of probiotics and propolis on plaque index, plaque pH, and salivary pH values. Journal of IMAB 31:6159-6165.
4. Darwita RR, Setijono K, Almasyhur F, Andreas P, Djais AA (2020) A comparative evaluation of the number of *Streptococcus mutans* colonies in the enamel surface of teeth after the application of CPP-ACP containing propolis and CPP-ACP without propolis. International Journal of Applied Pharmaceutics 12:69-72. https://doi.org/10.22159/ijap.2020.v12s2.PP-25.
5. Iswari AP, Riyanti E, Hadidjah D (2010) Plaque index differences before and after teeth brushing with and without propolis dentifrice. Padjadjaran Journal of Dentistry 22. https://doi.org/10.24198/pjd.vol22no1.14072.
6. Andiani SG, Darwita RR, Setiawati F (2019) The efficacy of propolis fluoride for arresting dentine caries in primary teeth. AIP Conference Proceedings 2092. https://doi.org/10.1063/1.5096747.
7. Darwita RR, Setiawati F, Andiani SG (2019) Association of propolis fluoride with arrested dentinal caries and dental plaque levels. Journal of International Dental and Medical Research 12:622-627.
8. De-Carli AD, Zárate-Pereira P, De-Carli G, Zafalon EJ, Zárate CBR, Yassumoto LM (2010) Ação da própolis de apis mellifera associada ao fluoreto de sódio sobre o biofilme dental: Ensaio clínico duplo cego randomizado. Rev Odontol Bras Central 19:310-313. https://doi.org/10.36065/robrac.v19i51.523.
9. Duailibe SA, Goncalves AG, Ahid FJ (2007) Effect of a propolis extract on Streptococcus mutans counts in vivo. J Appl Oral Sci 15:420-423. https://doi.org/10.1590/s1678-77572007000500009.
10. El-Allaky HS, Wahba NA, Talaat DM, Zakaria AS (2020) Antimicrobial Effect of Propolis Administered through Two Different Vehicles in High Caries Risk Children: A Randomized Clinical Trial. J Clin Pediatr Dent 44:289-295. https://doi.org/10.17796/1053-4625-44.5.1.
11. El-Allaky HS, Wahba NA, Talaat DM, Zakaria AS (2020) Evaluation of antimicrobial effect of two propolis formulae on *Streptococcus mutans* and *Candida albicans:* a randomized clinical trial. Alexandria Dental Journal 45:99-103. https://doi.org/10.21608/adjalexu.2020.88445.
12. Ercan N, Erdemir EO, Ozkan SY, Hendek MK (2015) The comparative effect of propolis in two different vehicles; mouthwash and chewing-gum on plaque accumulation and gingival inflammation. Eur J Dent 9:272-276. https://doi.org/10.4103/1305-7456.156851.
13. Finisha AR, Darwita RR, Amalliah I (2019) Relationship between oral hygiene and the activity of dentinal caries after applying propolis fluoride on primary teeth. AIP Conference Proceedings 2092. https://doi.org/10.1063/1.5096749.
14. Gautam N, Ahmed SO, Thapliyal D, Dingal S (2023) Clinical Validation of the Efficacy of a Mouthwash Comprising Propolis for the Prevention and Treatment of Plaque and Gingivitis: A Phase II Trial. J Oral Health Dent Res 3:1-6.
15. Hegde KS, Bhat SS, Rao A, Sain S (2013) Effect of Propolis on Streptococcus mutans Counts: An in vivo Study. Int J Clin Pediatr Dent 6:22-25. https://doi.org/10.5005/jp-journals-10005-1180.
16. Khabazian A, Mirhashemi FS, Sadeghi F (2025) Investigating the effect of propolis-containing chewing gum in comparison with propolis-containing mouthwash on reducing gingival inflammation in patients with gingivitis. BMC Oral Health 25:231. https://doi.org/10.1186/s12903-025-05564-8.
17. Kumar A, Sunkara MS, Pantareddy I, Sudhakar S (2015) Comparison of Plaque Inhibiting Efficacies of Aloe Vera and Propolis Tooth Gels: A Randomized PCR Study. J Clin Diagn Res 9:ZC01-03. https://doi.org/10.7860/JCDR/2015/13185.6413.
18. Mohsin S, Manohar B, Rajesh S, Asif Y (2015) The effects of a dentifrice containing propolis on Mutans Streptococci: a clinico-microbiological study. Ethiop J Health Sci 25:9-16. https://doi.org/10.4314/ejhs.v25i1.3.
19. Neto EMR, Valadas LAR, Lobo PLD, Fernandes AMB, da Cruz Fonseca SG et al (2020) Dose-response Evaluation of Propolis Dental Varnish in Children: A Randomized Control Study. Recent Pat Biotechnol 14:41-48. https://doi.org/10.2174/1872208313666190826145453.

20. Pereira EM, da Silva JL, Silva FF, De Luca MP, Ferreira EF et al (2011) Clinical Evidence of the Efficacy of a Mouthwash Containing Propolis for the Control of Plaque and Gingivitis: A Phase II Study. Evid Based Complement Alternat Med 2011:750249. https://doi.org/10.1155/2011/750249.
21. Siqueira ÉC, Campos FMT, Mendes JC, Lima LND, Araújo RS et al (2021) Evaluation of the Effects of Propolis and Xylitol Chewable Tablets on the Salivary Concentrations of Oral Microorganisms in Orthodontic Patients: A Pilot Study. J Young Pharm 13:68-71.
22. Sukmawati AN, Wijayanti P, Karina VM, Lastianny SP (2021) 10% propolis as a subgingival irrigation agent after gingival curettage: A comparative study. Journal of International Oral Health 13:571 - 578.
23. Tulsani SG, Chikkanarasaiah N, Siddaiah SB, Krishnamurthy NH (2014) The effect of Propolis and Xylitol chewing gums on salivary Streptococcus mutans count: a clinical trial. Indian J Dent Res 25:737-741. https://doi.org/10.4103/0970-9290.152182.
24. Lisbona-González MJ, Muñoz-Soto E, Reyes-Botella C, Olmedo-Gaya MV, Diaz-Castro J, Moreno-Fernandez J (2021) Study of the Antimicrobial Effect of an Ethanolic Extract of Propolis in Periodontal Disease. Applied Sciences 11:7463.
25. Sengupta U, Sogi HPS, Shah SG, Bala R, Saini R et al (2025) Comparative evaluation of antimicrobial effectiveness of 2% chlorhexidine, 25% aqueous extract of propolis, and 2.4% curcuma longa as intracanal irrigants: An ex vivo study. J Indian Soc Pedod Prev Dent 43:425-431. https://doi.org/10.4103/jisppd.jisppd_133_25.
26. Chandrashekaraiah P, Benzigar R, Shivananjan R, Arockiam S, Nandhini N, Ulaganathan S (2025) Comparative evaluation of propolis, fluoride and probiotic mouthwashes on streptococcus mutans and oxidative stress in fixed orthodontic patients: A triple-blind, randomized controlled trial with 9-month follow-up. Int Orthod 23:101017. https://doi.org/10.1016/j.ortho.2025.101017.
27. Mohammed NH, Abi-Elhassan MH, Safy RK (2025) Effect of Aloe Vera, Propolis and Pomegranate Extract Mouth Washes on Salivary pH and Antibacterial Activity in High Caries Risk Patients: A Randomized Controlled Clinical Trial. Afr J Bio Sc 7. https://doi.org/10.48047/AFJBS.7.9.2025.306-319.
28. Gispert Abreu E, Cantillo Estrada E, Rivero López A, Padrón Illance M (2000) Actividad anticaries de una crema dental con propóleos. Revista Cubana de Estomatología 37:166-170.
29. Naggar RAE, Ibrahim SH, Mosallam RS, Khairy MA (2021) Effect of Propolis and Pomegranate Extract Mouthwashes on Taste Alteration, Salivary pH and Antibacterial Activity in High Caries Risk Patients: A Randomized Control Trial. Indian Journal of Public Health Research & Development 12:463-470. https://doi.org/10.37506/ijphrd.v12i3.16105.
30. Anani H, Elasser D, Niazy M, Jamil W, Elsharkawy D (2023) Evaluation of the remineralization and antibacterial effect of natural versus synthetic materials on deep carious dentin: A randomized controlled trial. Dent Med Probl 60:87-97. https://doi.org/10.17219/dmp/147075.
